# A magnetic resonance multi-atlas for the neonatal rabbit brain

**DOI:** 10.1016/j.neuroimage.2018.06.029

**Published:** 2018-10-01

**Authors:** Sebastiano Ferraris, Johannes van der Merwe, Lennart Van Der Veeken, Ferran Prados, Juan-Eugenio Iglesias, Andrew Melbourne, Marco Lorenzi, Marc Modat, Willy Gsell, Jan Deprest, Tom Vercauteren

**Affiliations:** a Translational Imaging Group, Centre for Medical Image Computing (CMIC), Department of Medical Physics and Biomedical Engineering, University College London, Malet Place Engineering Building, London, WC1E 6BT, UK; b Department of Development and Regeneration, Woman and Child Cluster, Group Biomedical Sciences, KU Leuven University of Leuven, Belgium; c moSAIC Facility, Biomedical MRI, Department of Imaging and Pathology, KU Leuven, Belgium; d Université Côte d’Azur, Inria, France; e Wellcome/EPSRC Centre for Interventional and Surgical Sciences, University College London, UK; f Institute for Women's Health, University College London, UK

**Keywords:** Newborn rabbit brain animal model, MRI multi-atlas, Multi-atlas based segmentation

## Abstract

The rabbit model has become increasingly popular in neurodevelopmental studies as it is best suited to bridge the gap in translational research between small and large animals. In the context of preclinical studies, high-resolution magnetic resonance imaging (MRI) is often the best modality to investigate structural and functional variability of the brain, both *in vivo* and *ex vivo*. In most of the MRI-based studies, an important requirement to analyze the acquisitions is an accurate parcellation of the considered anatomical structures. Manual segmentation is time-consuming and typically poorly reproducible, while state-of-the-art automated segmentation algorithms rely on available atlases. In this work we introduce the first digital neonatal rabbit brain atlas consisting of 12 multi-modal acquisitions, parcellated into 89 areas according to a hierarchical taxonomy. Delineations were performed iteratively, alternating between segmentation propagation, label fusion and manual refinements, with the aim of controlling the quality while minimizing the bias introduced by the chosen sequence. Reliability and accuracy were assessed with cross-validation and intra- and inter-operator test-retests. Multi-atlas, versioned controlled segmentations repository and supplementary materials download links are available from the software repository documentation at https://github.com/gift-surg/SPOT-A-NeonatalRabbit.

## Highlights

• In magnetic resonance image analysis accurate automatic segmentations tools are critically important.
• Multi-atlas based methods are valuable tools to obtain an automatic MRI segmentation.
• The rabbit model has become increasingly popular in neuro developmental studies.
• Currently there is no multi-atlas of the newborn rabbit available in literature.
• In this manuscript we provide the first high resolution MRI multi atlas for the newborn rabbit brain.

## Introduction

Animal models are a crucial component for the advancement of neuroscience and although rodent neurocognitive models are well established, their translation value is limited, especially in view of their prenatal myelination, lissencephalic brain structure and low proportion of white matter (Bassan et al., 2000). As an alternative, rabbits can provide a link between small and large animals, as their brain develops in the perinatal period and their timing of white matter maturation is comparable to human (Derrick et al., 2004, 2007; Eixarch et al., 2012; Drobyshevsky et al., 2007). Additionally, the rabbit brain has a more complex structure than rodents with a higher white matter proportion, and low circulating levels of xanthine oxidase (White et al., 1996). Yet, the more complex brain structure does not come at the cost of large animal models that are also primarily prenatal brain developers.

Digital atlases are a widely used tool for most studies related to volume, shape and microstructure quantification of specific brain regions (Toga and Thompson, 2000). To the best of our knowledge, a neonatal rabbit brain atlas has not been published or made available. Its best approximation available in the literature, is the adult rabbit digital multi-atlas proposed by Muñoz-Moreno et al. (2013). This is based on 10 multi-modal scans acquired at 3 T with anisotropic resolution (0.15 mm, 0.15 mm, 0.7 mm) and it provides 60 anatomical regions, whose borders were identified by overlaying structural and diffusion weighted imaging (DWI) derived modalities. In Müllhaupt et al. (2015), the authors proposed an anatomical delineations of the coronal slices of the adult rabbit, however, these segmentations are currently not available in digital format.

The lack of a digital atlas prevents from applying the state-of-the-art automatic methods. This is particularly critical when considering the growing range of studies involving neonatal rabbits, especially in the investigation of pre-term birth related perinatal brain injury (Lei et al., 2017), cerebral palsy (Tan et al., 2005; Derrick et al., 2007; Drobyshevsky and Quinlan, 2017) and intrauterine growth restriction (Batalle et al., 2014; Simões et al., 2015).

The preferred strategy to obtain a segmentation is through manual delineation based on multiple MR modalities (Eixarch et al., 2012; Drobyshevsky et al., 2012; Lim et al., 2015). This choice allows for direct control of the results quality. Yet, it is intrinsically biased by inter- and intra-operator variability, dependent upon training skills and impractical for large studies because it is very time consuming. Atlas-based approaches dramatically reduce the work load and make the segmentation reproducible, although their accuracy improvement remains an active area of research.

When a comprehensive MR atlas or a probabilistic atlas (obtained by co-registering and averaging together several atlases) is available, it can be transposed over the image awaiting to be parcellated, with a segmentation propagation method (Pham et al., 2000). In a similar approach, the image anatomy can be transposed in the space of the atlas (Ashburner and Friston, 2005).

Accuracy can be further improved by a multi-atlas based approach (Iglesias and Sabuncu, 2015), hereby the propagation and fusion from multiple atlases can better span the inter-subject variability (Heckemann et al., 2006; Cardoso et al., 2013; Sabuncu et al., 2010; Aljabar et al., 2009; Koch et al., 2017). In addition, to enhance the segmentation propagation robustness, a multi-modal registration method can be employed in the pipeline (Wang et al., 2013; Ma et al., 2014). Ultimately, different segmentation methods can be as well combined, i.e. a manual refinement can be applied to an initial segmentation obtained with an automatic algorithm.

In this paper we present the first micro-MR multi-modal multi-atlas for the neonatal rabbit brain, delineating 89 areas for 12 subjects. Alongside we provide an open-source segmentation propagation and label fusion algorithm. Combined together they serve as a tool for the automatic segmentation of the newborn rabbit brain.

## Materials and methods

### Animal preparation and sample collection

**Fig. 1:**
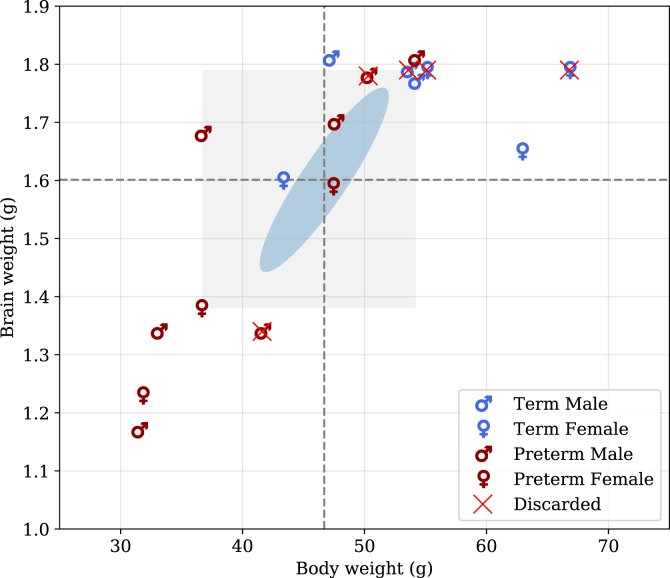
**Sample selection and distribution.** Brain and body weight of all subjects prepared for the study: 8 pre-term (7 males/3 females) and 4 term (3 males/4 females). Of the initial 17 subjects (10 pre-term, 7 term), 5 have been discarded due to image artefacts. Cross-hair shows the mean, blue ellipsoids the covariance and gray rectangle shows the $25^{\text{th}}$ and $75^{\text{th}}$ percentiles.

The proposed atlas was produced as part of a wider study, evaluating encephalopathy of prematurity in a pre-term newborn rabbit model. Initially, 17 subjects, both born either term (gestation 31 days) or pre-term (gestation 28 days), were prepared according to the following protocol (see Fig. 1):

1. Time-mated pregnant does (hybrid of New Zealand White and Dendermonde) were obtained from the *Animalium* of the Biomedical Sciences group at the KU Leuven, Belgium. Animals were treated according to current guidelines for animal well-being, and all experiments were approved by the Ethics committee for Animal Experimentation of the Faculty of Medicine (P062/2016). Does were housed in separate cages before delivery, with free access to water and chow and a light-dark cycle of 12 h. The does underwent a cesarean section on either 28 (pre-term) or 31 (term) days of gestation. Thereafter, newborn rabbits were nursed in an incubator with twice daily gavage feeding until harvesting on post-conceptional age of 32 days. This established an equivalent time-point at harvesting for both term (gestation 31 days + 1 day postnatal) and pre-term (gestation 28 days + 4 days postnatal).
2. Neonatal rabbits were actively perfused with a mixture of formalin and gadolinium to increase MRI signal to noise ratio while preserving the tissue (Johnson et al., 2002). Kits were anesthetized with intramuscular ketamine (35 mg/kg, ketamine 1000 CEVA; CEVA Sant Animal, Brussels, Belgium) and xylazin (6 mg/kg, Vexylan; CEVA Sant Animal) and transcardially perfused with 0.9% saline and heparin (100u/mL) followed by perfusion fixation with 4% paraformaldehyde in 0.1 mol/L phosphate buffer (pH 7.4) containing dimeglumine gadopentetate 0.5 mmol/mL (Magnevist ^®^, Dimeglumine Gadopentetate 0.5 mmol/mL, Bayer HealthCare Pharmaceuticals, Germany).
3. The head with the brain *in situ* was then immersed in this solution for another 48 h followed by a rehydration phase in a 1:200 solution of Magnevist ^®^/PBS for 48–72 h. The head was trimmed and placed in a sample holder, surrounded by proton-free perfluoropolyether solution (Fomblin ^®^, Solvay Solexis) that minimizes susceptibility artefacts at the interface.

### Micro-MRI and DWI acquisition

Micro-MRI scans (voxels with resolution in the order of 100μm) were performed using a Bruker Biospec 9.4 T small animal MR scanner (Bruker Biospin, Ettlingen, Germany; horizontal bore, 20 cm) hosted at the Department of Imaging and Pathology, KU Leuven, Belgium. It was equipped with actively shielded gradients (600 mT/m) utilising a rat brain surface receiver decoupled to a volume quadrature transmit coil (internal diameter of 72 mm).

▷ Structural T1 weighted image acquisition: Three-dimensional MR images were acquired using a gradient-echo sequence (3D Flash) with Time of Echo (TE)/Time of Repetition (TR) 5.5/50 ms; flip angle 70°; bandwidth 50 kHz; acquisition matrix of 320×385×230; isotropic resolution 78×78×78μm; 4 averages; acquisition time 1h20 min.
▷ Diffusion-weighted image acquisition: After the T1 acquisition, diffusion-weighted images (DWI) were acquired using a 3D segmented spin-echo version of the echo-planar imaging (SE-EPI) sequence with 8 segments; TR/TE: 280/24 ms; FOV: 30×25×25mm; matrix: 192×160×160 conferring an isotropic spatial resolution of 156μm; 64 directions and 3 b-values per direction ($800,1000,1500\;\text{s}/{\text{mm}}^{2}$); acquisition time 10 h.

### Image processing pipeline and proposed stereotaxic orientation

Acquired data were converted from Bruker ParaVision format to Nifti-1 format using the *bruker2nifti* image converter (Ferraris et al., 2017). Despite being customary for rats, the bicommissural orientation (where the anatomical plane passing through the anterior and posterior commissure is parallel to the horizontal plane) is not applicable for rabbits. See supplement A for further comparisons between rat and rabbit.

**Fig. 2:**
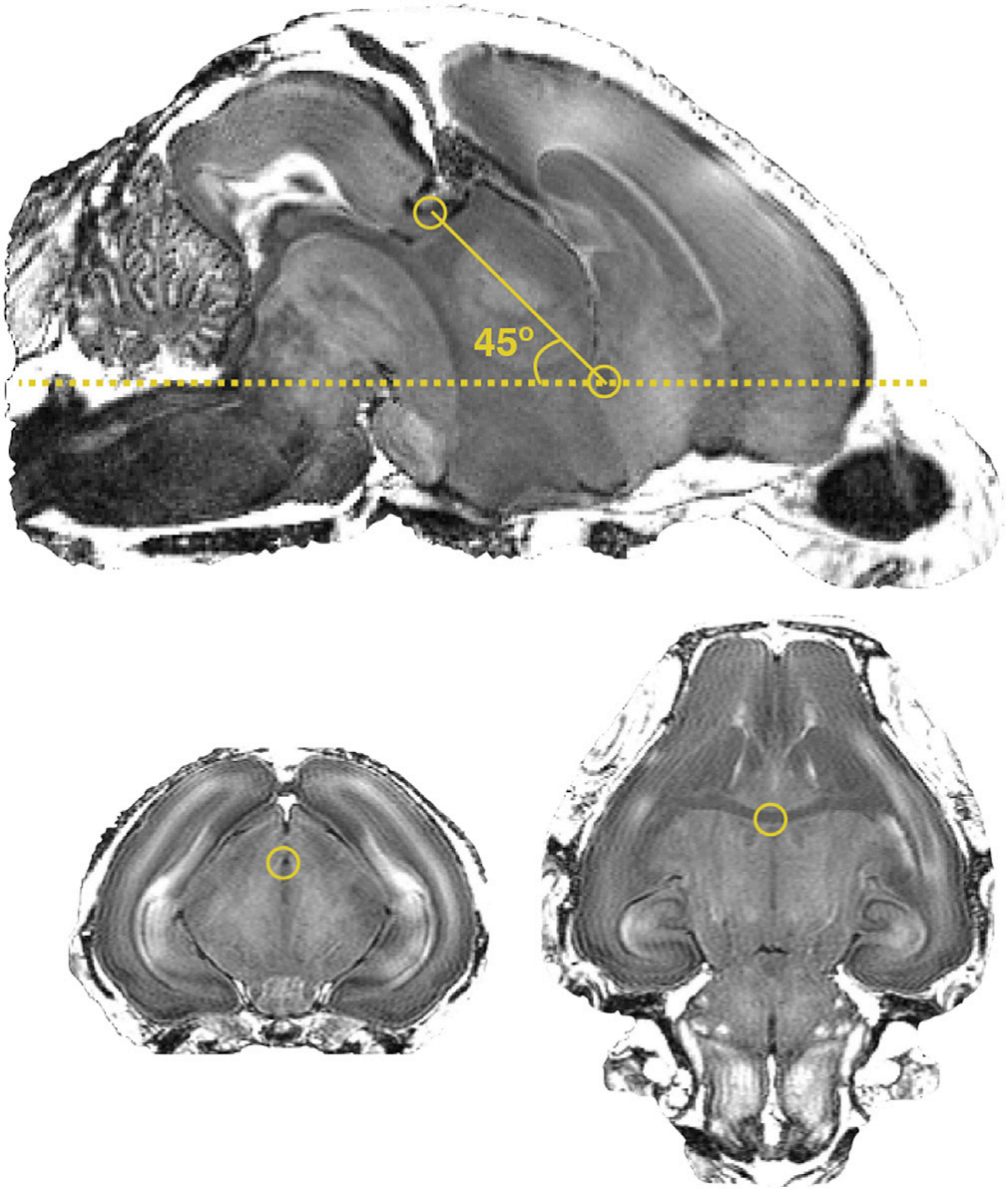
**Proposed stereotaxic orientation for the MR imaged newborn rabbit brain.** In the mid-sagittal section, the bicommissural plane forms a $45^{\circ }$ angle with the horizontal plane. The full line represents the bicommissural plane and the dashed line the horizontal plane. Centers of the anterior and posterior commissure are highlighted in the coronal and the axial sections.

To attain a coronal plane comparable to histological atlases, not based on skull landmarks, that are not suitable for the developing brain (Shek et al., 1986; Pivik and Braun, 1978), we propose a stereotaxic orientation where the bicommissural plane forms a constant angle of $45^{\circ }$ with the plane parallel to the horizontal plane (represented in Fig. 2 with full and dashed lines respectively). In this coordinate system, the ventral side of the brain is aligned with the horizontal plane.

After subjective visual evaluation of bias field and artefacts, the best acquisition underwent manual alignment in stereotaxic orientation. Thereafter, brain and skull coarse region of interest manual delineation was performed by alternating Gaussian smoothing and symmetrisation with respect to the mid-sagittal plane employing *ITK-SNAP* and *NiftySeg* (Yushkevich et al., 2006; Cardoso et al., 2012).

The binary mask isolating the region of interest was then automatically propagated on the T1 modality of the remaining 11 acquisitions with an affine segmentation propagation performed with *NiftyReg* (Modat et al., 2014). After the initial orientation and the coarse brain and skull extraction for all of the subjects, the *N4ITK* bias field correction (Tustison et al., 2010) was applied. An additional mask was created by thresholding the outliers intensities while manually selecting the threshold percentile. This was subsequently employed to mitigate the impact of image artefacts during the spatial alignment. Both masks were propagated to the B0 weighted image before eddy current correction and tensor fitting, performed with *FSL* (default parameters) version 5.0.9 (Jenkinson et al., 2012).

Fractional anisotropy map (FA), mean diffusivity map (MD) and RGB encoded direction of the main eigenvalue map (V1) were resampled in the T1 weighted image space to overlay structural information. Due to sample as well as frequency drifting occurred during the acquisition, anatomies resulting from different sequences were not perfectly spatially aligned. Therefore the B0 was rigidly aligned with the T1 (Normalized Mutual Information as measure of similarity) and the same transformation was applied to the remaining DW modalities.

### Manual segmentation and taxonomy

The available adult atlas (Muñoz-Moreno et al., 2013) could not be satisfactory propagated onto the neonatal rabbit anatomy, mainly due to the geometrically non-linear growth between newborn and adult. After a manual landmark based non-rigid registration with 3D Slicer Kikinis et al. (2014), the adult atlas was roughly deformed over the newborn anatomy, and considered as a starting point. The subsequent manual adjustment had to compensate for the anatomical differences and for the ringing artefacts caused by resampling (adult grid spacing: 0.15×0.15×0.7mm, newborn grid spacing: 0.078×0.078×0.078mm).

**Fig. 3:**
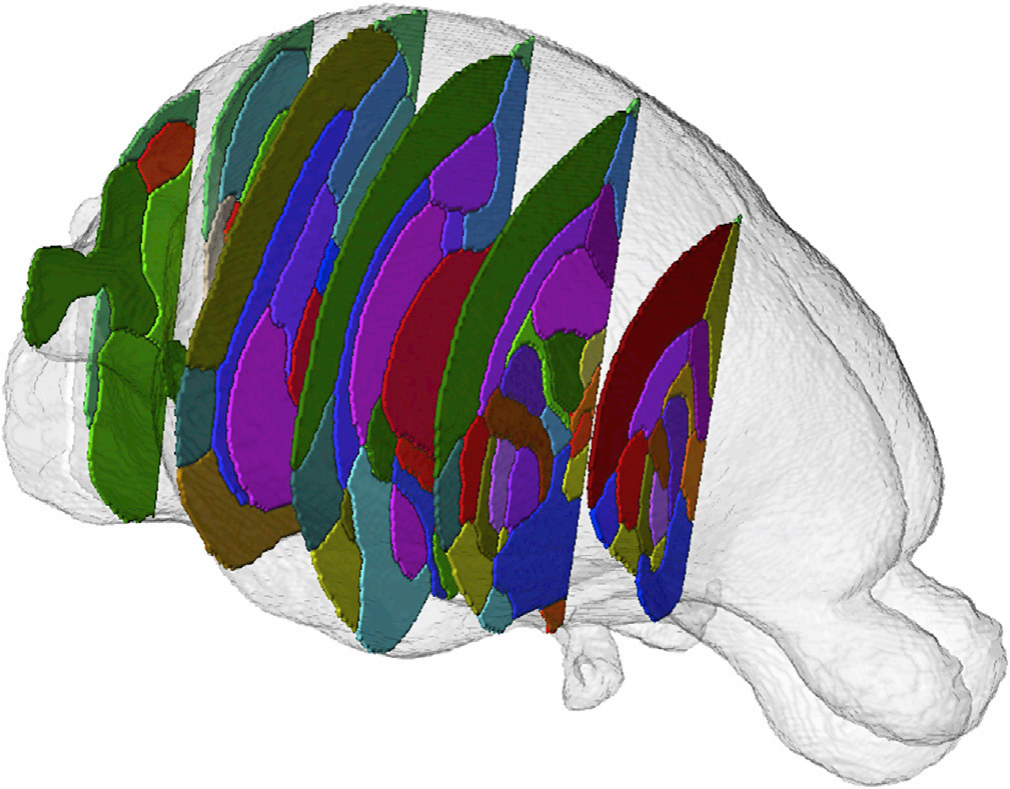
**3D brain surface rendering.** Segmentation of 6 selected coronal slices of the right hemisphere are shown with the remaining labels in transparency. Corresponding slices are delineated over the T1 modality with the corresponding nomenclature in Fig. 4.

**Fig. 4:**
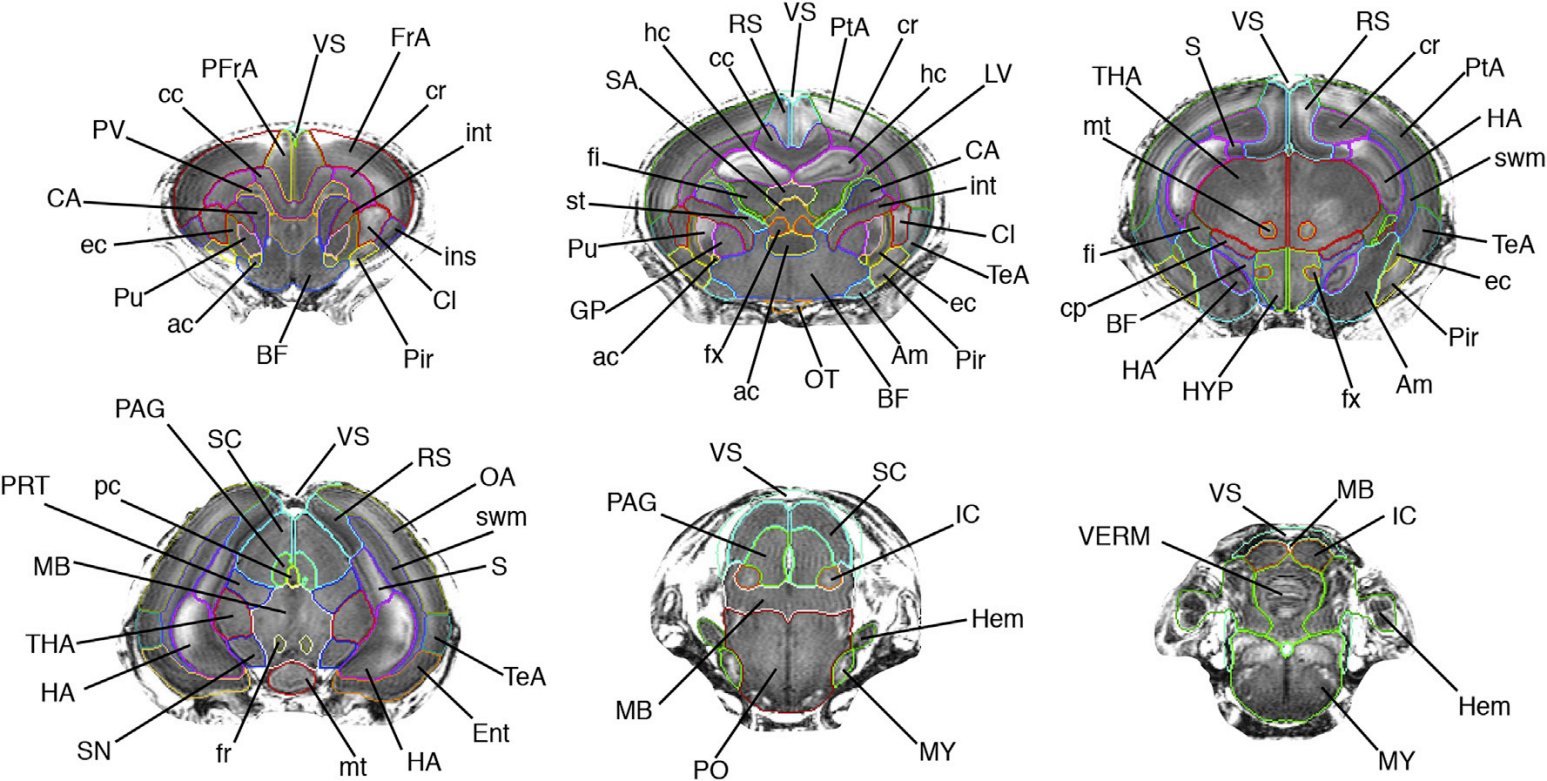
**Delineation of anatomical regions over the T1 modality.** Progressive coronal sections, anterior to posterior. Only the segmentation outline is delineated for visualization purposes. Region of interest surrounding the brain is visible in the image, to show the border delineation between the brain and the skull. See Table 1 for the abbreviation and nomenclatures. Detailed taxonomy table is proposed in the additional material.

To reduce the complexity of the manual segmentation, one hemisphere was refined and subsequently registered on the contralateral side (Ranzini et al., 2017) providing a starting point for further adjustment. Manual refinement was performed by author JvdM using *ITK-SNAP* v2.2.0 (Yushkevich et al., 2006), overlaying the T1, FA, MD and V1 modalities. The neuroanatomical nomenclature selected was adapted from a standard adult rabbit histological atlas (Shek et al., 1986) and from two standard rat brain atlases (Paxinos George, 2007; Swanson, 2004), incorporating 89 labels (Table 1 and Fig. 3, Fig. 4). Areas requiring histological criteria for identification were not detailed in the atlas. A hierarchical taxonomy inclusive of the areas appearing in the histology and the produced manual protocol are provided in the supplementary material B.

**Table 1:** **Delineated regions in the proposed new-born rabbit brain taxonomy.** For symmetric structures, we followed the convention left/right odd/even. An extended hierarchical taxonomy is provided in the supplementary material B.

Level	Region (abbrev)	Label
1.1	Frontal Area (FrA)	7, 8
	Medial Prefrontal (PFrA)	5, 6
	Occipital Area (OA)	9, 10
	Parietal Area (PtA)	11, 12
	Temporal (TeA)	13, 14
	Cingulate (Cg)	15, 16
	Retrosplenium (RS)	17, 18
	Insular (Ins)	19, 20
1.2	Olfactory lobe (OB)	25, 26
	Piriform (Pir)	27, 28
1.3	Hippocampus (HA)	31, 32
	Subiculum (S)	43, 44
	Entorhinal (Ent)	45, 46
1.4	Claustrum (CL)	53, 54
	Amygdala (Am)	55, 56
1.5	Caudate nucleus (CA CN)	69, 70
	Putamen (Pu)	71, 72
	Globus Pallidus (GP)	75, 76
	Basal forebrain (BF)	77
	Septum (SA)	78
2.1	Thalamus (THA)	83, 84
	Hypothalamus (HYP)	109, 110
	Mammillary body (MAM)	121
2.2	Midbrain (MB)	127
	Pretectal (PRT)	129, 130
	Superior colliculus (SC)	133, 134
	Inferior colliculus (IC)	135, 136
	Substantia nigra (SN)	139, 140
	Periaqueductal gray (PAG)	141, 142
2.3	Pons (PO)	151
	Medulla oblongata (MY)	153
3.1	Cerebellar vermis (VERM)	161
3.2	Cerebellar hemisphere (HEM)	179, 180
4	Ventricular system (VS)	201
	Lateral ventricles (LV)	203, 204
	Periventricular area (PV)	211, 212
5.1	Optic tract and optic chiasm (OT)	215
5.2	Corpus callosum (cc)	218
	External capsule (ec)	219, 220
	Internal capsule (int)	223,224
	Corona radiata (cr)	225, 226
	Cerebral peduncle (cp)	227, 228
	Subcortical white matter (swm)	229, 230
5.3	Anterior commissure (ac)	233
	Hippocampal commissure (hc)	237
	Fimbria of hippocampus (fi)	239, 240
	Columns of the fornix (fx)	241, 242
	Stria terminalis (st)	243, 244
5.4	Mammilothalamic tract (mt)	247,248
	Fasciculus retroflexus (fr)	251,252
	Posterior commissure (pc)	253

**Fig. 5:**
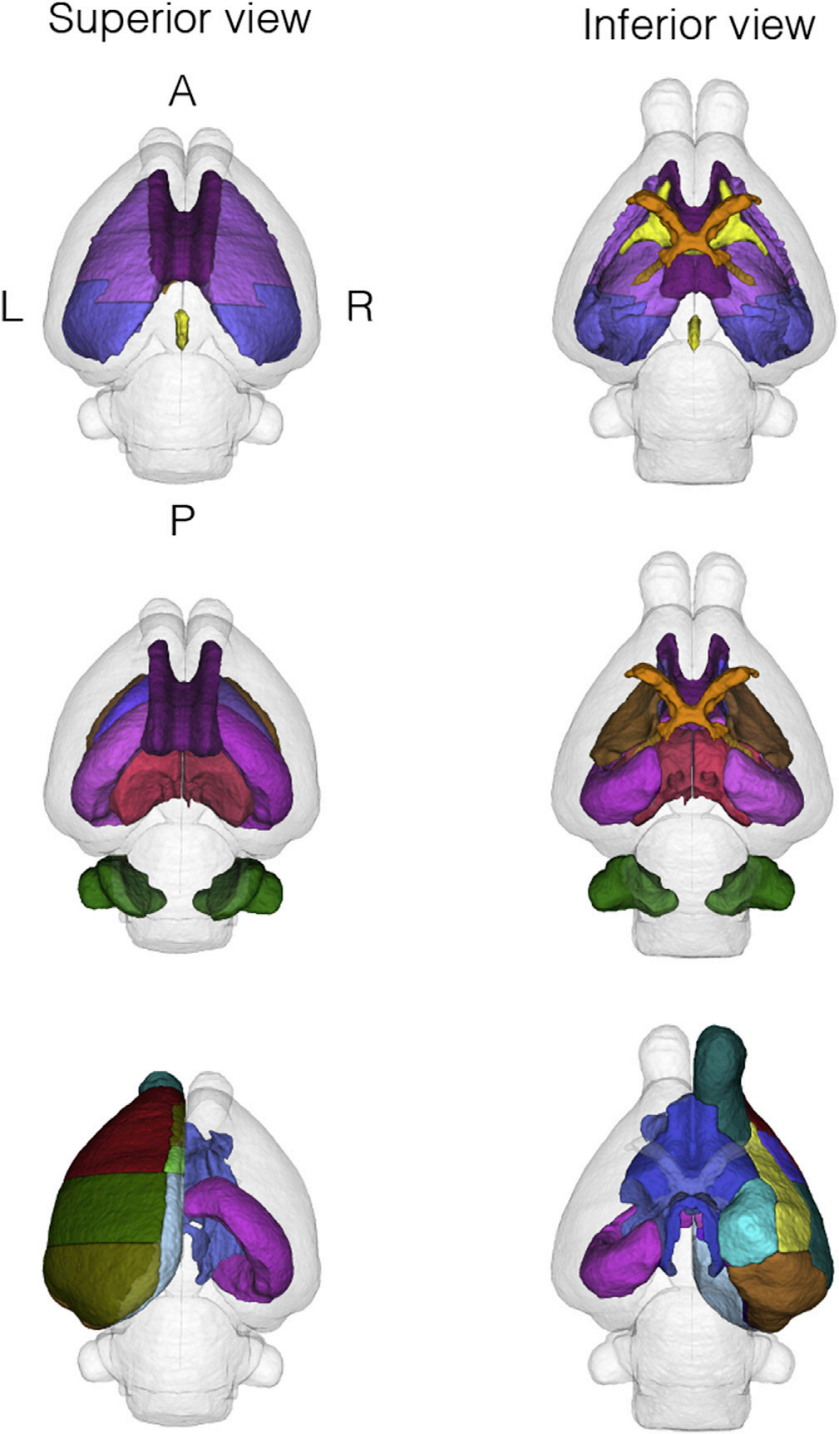
**3D brain surface rendering.** The first row emphasizes a selection of subcortical regions (corona radiata in pink, corpus callosum in purple and subcortical white matter in blue) and of fibertracts (anterior and posterior commissure in yellow, and optic tracts in orange). The second row highlights the regions that are believed to be affected by pre-term birth. Superior view: corpus callosum (purple), hippocampus left and righ (pink), thalami (red), caudate nuclei (blue) and cerebellar hemispheres (green). Inferior view: optic tracts (orange), internal capsula left and right (brown). The third row points out the right hemisphere cortex. Superior view: olfactory bulb (aquamarine blue), anterior (red), frontal (green) and occipital (khaki). Inferior view: piriform (yellow), enthorinal (brown) and amigdala (turquoise), the hippocampi (left and right in pink) and the basal forebrain (blue).

Delineations covered the whole brain up to the medulla oblongata posterior, partly incorporating some of the ventricular system while excluding the proximal spine and skull. Supplementary material C provides the manual delineation protocol.

Regions were classified into cortical regions, cortical subplate, basal ganglia, brainstem, cerebellum, fibertracts and ventricular system:

▷ Cortical and cortical subplate delineation was based upon T1 and V1 intensities, and were defined by their surrounding fibertracts and manually corrected for by neuroanatomical landmarks.
▷ The delineation of the basal ganglia was based on T1 weighted and FA, MD intensities while the brainstem with less distinctive T1 definition was based upon the combination of all modalities equally.
▷ The fibertracts delineation was mainly based on the T1 and FA intensities, and only secondarily on the V1.
▷ Finally, cerebellar hemispheres and vermis were almost completely segmented based on only T1 weighted characteristics.

After completing the manual refinement of the first subject, this was propagated to the next one via affine and non-rigid registration. Non-linear spatial correspondences were estimated through diffeomorphic registration of the bias field corrected T1 images (Modat et al., 2010). Subsequent to manual refinement, both segmentations were propagated to the third subject. Through visual assessment, the most suitable of the two propagations was subsequently manually refined. Thereafter, the diffeomorphic segmentation propagation of each of the three refined subjects on the new one to be segmented, was followed by the application of four label fusions methods. This was performed both on the T1 modality alone, or on the T1 and FA combined, in a multi-modal approach. *Majority voting*, *Simultaneous Truth And Performance Level Estimation (STAPLE)* (Warfield et al., 2004) and *Similarity and Truth Estimation for Propagated Segmentations (STEPS)* (Cardoso et al., 2013), with a range of parametrizations selected through visual assessment, were applied to provide 8 mono-modal and 8 multi-modal starting points for a further manual refinement.

Majority voting is the simplest label fusion method. When applied, the final label selected for each voxel is the one that have appeared more often when propagating the segmentation of each subject in the multi-atlas. STAPLE estimates the value of the final label adding at the spatial distribution of the segmented structures a spatial homogeneity constraint within a probabilistic model optimized with an Expectation-Maximisation algorithm. STEPS extends STAPLE, considering a ranking strategy based on the local normalized cross correlation (LNCC) measure of similarity applied to the propagated anatomies on the new subject anatomy.

**Fig. 6:**
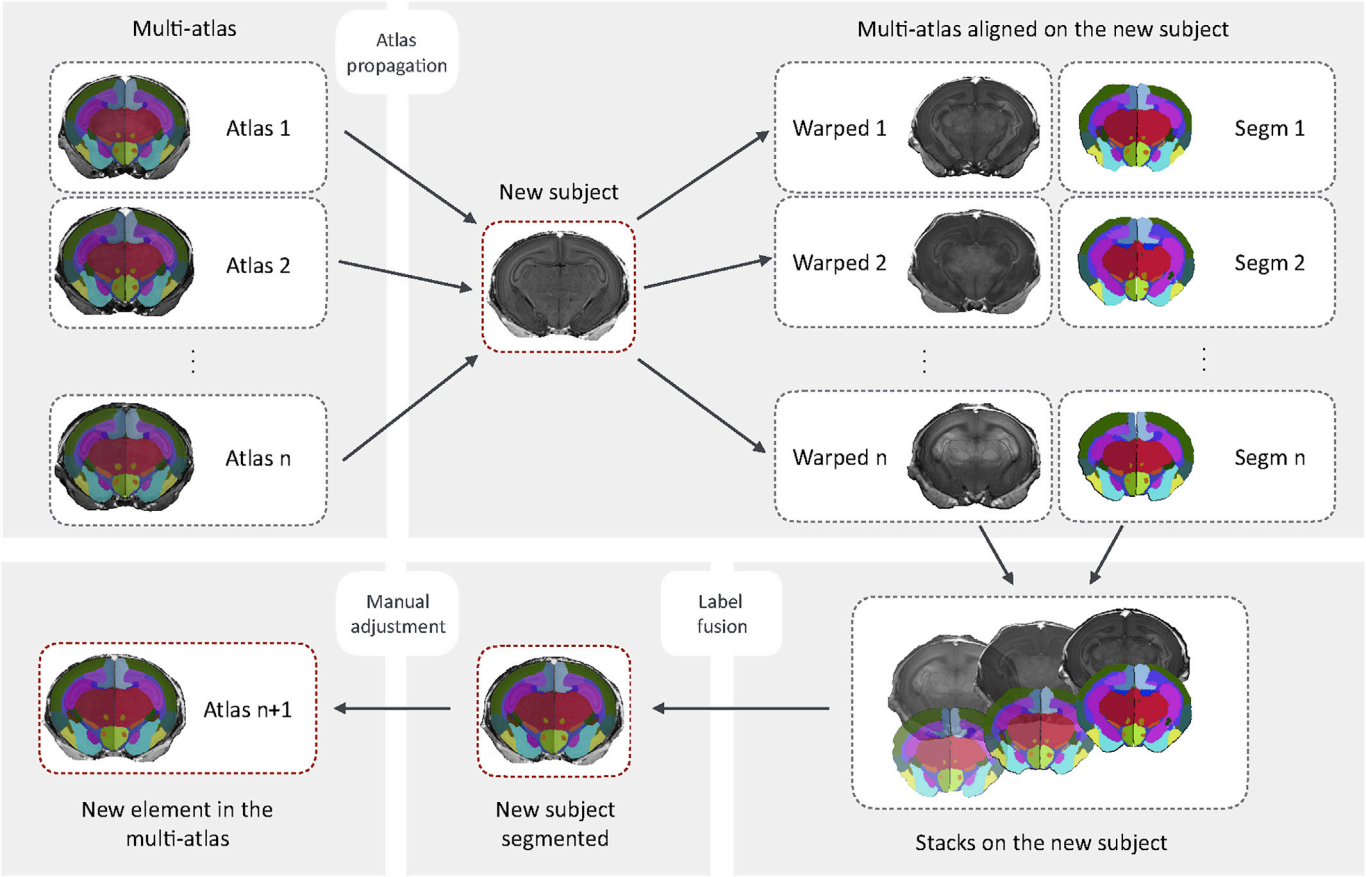
**Software scheme.** Visualization of the segmentation procedure, involving atlas propagation, label fusion and elective manual adjustments. For the creation of our multi-atlas the whole procedure with the manual adjustments had been performed three times.

With the proposed iterative method, schematically represented in Fig. 6, each new subject included to the multi-atlas improved the accuracy of the segmentation propagation and label fusion applied to the next one. To address the bias introduced when selecting an initial subject and to gradually improve the overall quality, the iterative method was applied again twice over the already segmented subjects.

The time of the manual adjustment has decreased significantly, from a time range between 20 and 25 h for the first subject to a time range between two to 6 h, depending on the outcome of the propagation.

### Automatic segmentation algorithm

The same procedure developed to initialize the segmentations for the manual adjustment can be extended to approximate the segmentation on a new subject, constituting an automatic segmentation algorithm. The first step consists in the diffeomorphic registration of the region of interest (brain and skull) mask with the subsequent propagation of the labels from each element of the multi-atlas to the new subject.

Among the several label fusion methods employed to produce the resulting parcellation from the stack of the aligned elements of the multi-atlas, the multi-modal Majority Voting provided the best result, both scored with visual assessment and leave-one-out cross validation. Accordingly, this is the proposed choice for the automatic segmentation method.

### Probabilistic atlas creation

**Fig. 7:**
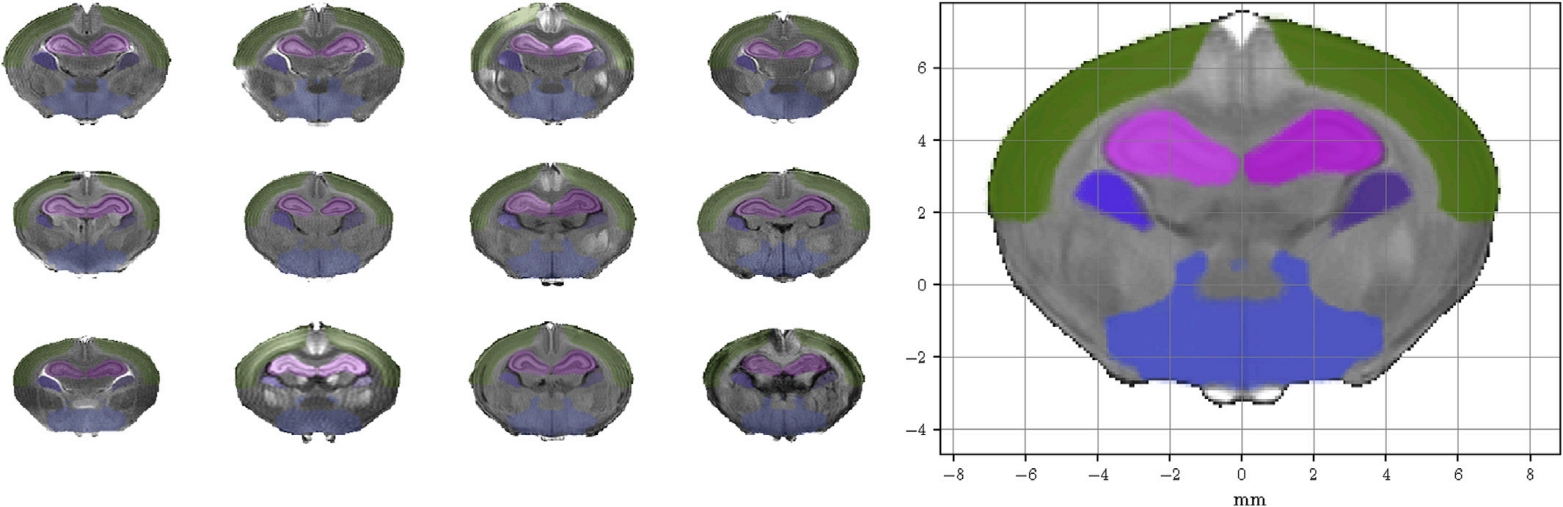
**Probabilistic atlas and probabilistic segmentation of 4 selected regions.** Coronal sections at the origin of the coordinate system for each of the 12 subject of the multi-atlas. Each subject is shown with the manual segmentation of parietal cortex (green), hippocampi (pink), caudate nuclei (purple) and basal forebrain (blue). On the right side, the probabilistic atlas as average of the 12 subjects on the left, with the voxel-wise probabilistic labels shown in the space of the atlas for each of the mentioned regions.

A probabilistic atlas can provide a suitable representation of the average newborn rabbit brain as well as a reference for future studies (Mazziotta et al., 1995). Fig. 7 shows an axial section of the neonatal rabbit brain created with an unbiased group-wise registration applied to the skull stripped T1 acquisitions with *NiftyReg* (Modat et al., 2012). The deformation model consists of diffeomorphisms parameterized with stationary velocity fields (Arsigny et al., 2006; Vercauteren et al., 2007).

## Results

Of the initial seventeen subjects to be segmented, visualized in Fig. 1, five had to be discarded due to the presence of enlarged perivascular spaces (n=1), most likely caused by the perfusion technique, ghosting artefacts (n=3) and wrong input scanner settings (n=1).

In a procedure involving automatic and manual steps, the remaining 12 subjects were parcellated into the 89 regions listed in Table 1. Illustrative views of one subject of the resulting multi-atlas can be visualized in Fig. 3, Fig. 4, were selected coronal sections are shown in 3D and annotated in the orthogonal 2D view. Superior and inferior views of the 3D rendering are proposed in Fig. 5, emphasising a selection of regions. The resulting software schematic employed to create the multi-atlas is illustrated in Fig. 6.

Computational time varies according to the computer architecture and the choice of the algorithm parameters. When the pipeline runs on a 2.5 GHz Intel Core i7 processor with SSD hard drive 16 GB 1600 MHz DDR3 and the affine and non-rigid registration steps are parallelised with OpenMP on 8 cores, the computational time for each automatic segmentation involving all the 12 subjects of the multi-atlas is approximately 2h10, including the full range of label fusion methods, as described in Section 2.4.

An approximation of the “ideal” average newborn rabbit brain in the proposed stereotaxic orientation is achieved with multimodal group-wise diffeomorphic registration. Its axial section passing through the origin of the coordinate system is shown in Fig. 7 besides the same slice for each of the 12 subjects of the multi-atlas. With the probabilistic atlas, it is possible to quantify the likelihood of the presence of a determined tissue type at a given voxel.

As called for by Mazziotta et al. (1995), the neonatal multi-atlas here proposed is intended as a starting point for a *maintained* digital atlas (https://github.com/gift-surg/SPOT-A-NeonatalRabbit). It is open to further improvements and to evolve according to future needs of structural and functional investigations. In analogy with what is currently employed in software development, possible further upgrades will be tracked in the hosting repository with a version control system.

## Validation

When the same protocol is applied, the manual segmentation variability between and within operators can be significant, even when segmenting a single region. Taking into account this known limitation, to assess the validity and robustness of an automatic method respect to the manual counterpart we performed five experiments:

1. Inter-operator variability assessment and comparison with the automatic method via hippocampi segmentation.
2. Visual scoring of unlabeled segmentations of the same subject.
3. Intra-operator consistency assessment for the manual adjustment of the automatic initialization via test-retest.
4. Fully automated segmentation assessment via cross validation.

The comparison of their outcomes provides an assessment of the coherence between the manual and the automatic approach. Before detailing them, we briefly recall the definitions of a range of measures to compare two segmentations (Taha and Hanbury, 2015; Herdin et al., 2005). These were selected before starting the investigation.

### Selected measures of similarity

As discussed in Subsection 6.3, the four selected measurements are aimed at obtaining a range of complementary quantifications.

*Dice's score:* it measures the number of overlapping voxels of two regions, over the mean of the voxels. It is defined as

$$\text{Dice}\left(A_{1},A_{2}\right)=\frac{2|A_{1}\cap A_{2}|}{\left|A_{1}|+|A_{2}\right|}\;\text{,}$$

where $\left|A_{j}\right|$ corresponds to the cardinality of the voxels of the region $A_{j}$.

*Covariance distance:* the segmented regions are considered as clouds of points, whose covariance matrices are compared. It provides an idea of how well the relevant features of the distributions are aligned when orthogonally translated onto the same centre, and it is defined as:

$$\text{CovDist}\left(A_{1},A_{2}\right)=\alpha \left(1-\frac{\text{Tr}\left(c\left(A_{1}\right)c\left(A_{2}\right)\right)}{\left||c\left(A_{1}\right)||+||c\left(A_{2}\right)|\right|}\right)\;\text{,}$$

where $c\left(A_{j}\right)$ is the covariance matrix of the voxel distribution of the label *j* in the 3D space, Tr is the matrix trace, or the sum of the diagonal elements, ||⋅|| is the Frobenius norm and *α* is a multiplicative factor corresponding to the maximal possible dissimilarity (here α=10).

*Symmetric Hausdorff distance:* it provides the maximal distance between the contour of one segmentation and the other one. It is defined as

$$\text{HD}\left(A_{1},A_{2}\right)=\text{max}\left(\left\{\text{H}\left(A_{1},A_{2}\right),\text{H}\left(A_{2},A_{1}\right)\right\}\right)\;\text{,}$$

for

$$\text{H}\left(A_{i},A_{j}\right)=\underset{a_{i}\in \partial A_{i}}{\text{max}}d\left(a_{i},\partial A_{j}\right)\;\text{,}$$

where $d\left(a_{i},\partial A_{j}\right)$ is the value of the minimal euclidean distance between the contour of $A_{j}$, indicated with $\partial A_{j}$, and the point $a_{i}$ belonging to $A_{i}$. The contour considered in this case is the layer of voxels delineating region shape $A_{j}$, still belonging to the region (internal contour).

*Normalized symmetric contour distance:* is a robust symmetric average of the mean of the minimal distances between the two segmentations for each voxel of the contour. It is defined as:

$$\text{NSCD}\left(A_{1},A_{2}\right)=\frac{\text{S}\left(A_{1},A_{2}\right)+\text{S}\left(A_{2},A_{1}\right)}{\left|\partial A_{1}|+|\partial A_{2}\right|}\;\text{,}$$

for

$$\text{S}\left(A_{i},A_{j}\right)=\sum_{a_{i}\in \partial A_{i}}d\left(a_{i},\partial A_{j}\right)\;\text{,}$$

with the same notations as defined above.

### Hippocampi manual segmentation comparison

A randomly selected and unlabeled subject was selected to undergo manual segmentation of the left hippocampus. This was performed by ${\text{rater}}_{1}$ (author JvdM) and ${\text{rater}}_{2}$ (author LVDV), following the same protocol established during this study and provided in the supplementary material C. ${\text{Rater}}_{1}$ performed the manual adjustments during each phase of the multi-atlas creation.

**Fig. 8:**
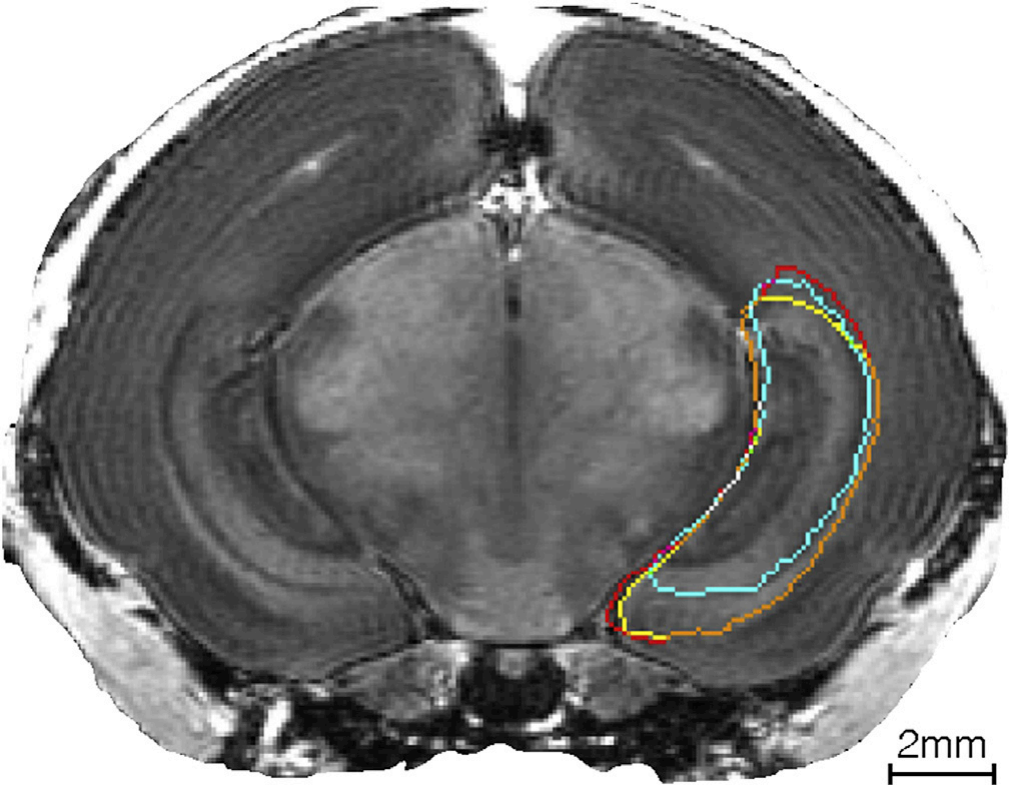
**Visual comparison of the inter-rater variability and automatic method in the segmentation of the left hippocampus.** Comparison between automatic method in red, ${\text{rater}}_{1}$ in yellow (expert) and ${\text{rater}}_{2}$ in turquoise (non expert) for the segmentation of hippocampi, in an axial slice. Regions of intersection between borders are ${\text{rater}}_{1}\cap \text{auto}$ in orange, ${\text{rater}}_{2}\cap \text{auto}$ in purple, ${\text{rater}}_{1}\cap {\text{rater}}_{2}$ in green and ${\text{rater}}_{1}\cap {\text{rater}}_{2}\cap \text{auto}$ in white.

Differences between the three regions are quantified with the four selected measures and reported in Table 3. A visual assessment of the differences is proposed in Fig. 8.

### Blinded segmentation scoring

The aim of this test was to compare the quality of the progressive manual refinements and secondarily to quantify the comparison between automatic and manual adjustment. A randomly selected and anonymized subject was considered with the three progressive rounds of manually refined segmentations (${\text{round}}_{1}$, ${\text{round}}_{2}$, ${\text{round}}_{3}$) and with the outcome of the automatic segmentation based on the remaining 11 subjects (auto). These four segmentations were relabelled, randomly re-ordered and provided to ${\text{rater}}_{1}$ for visual scoring. Visual scoring criteria to evaluate the goodness of a segmentation are subjective and based on the compatibility of the borders with the anatomical structures and on the overall smoothness.

The first of the manual adjustments, ${\text{round}}_{1}$, was scored as the worst one in term of quality and the latest manual adjustment, ${\text{round}}_{3}$, was scored as the best one. In between, ${\text{round}}_{2}$ and auto were considered equivalent in term of quality based only on blinded visual assessment.

### Consistency in the manual adjustment of the automatic parcellation assessment

To produce the multi-atlas, each subject underwent a series of three segmentation propagations and manual adjustments. In order to assess the accuracy of the automatic method and at the same time to measure intra-rater variability in the manual adjustment, a randomly selected and anonymized subject was automatically segmented using the remaining 11 subjects, and subsequently underwent manual adjustment twice, in a test-retest experiment.

**Fig. 9:**
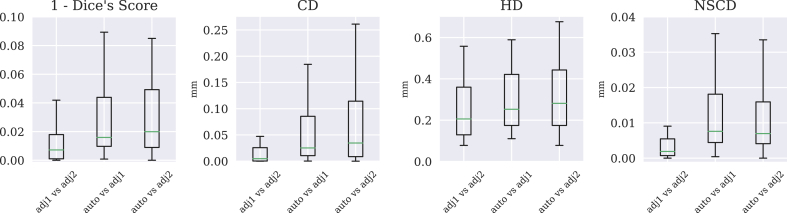
**Intra-rater test-retest.** Box plot comparing two different manual adjustments of the same initial segmentation (*adj1*, *adj2*) and the automatic initialization (*auto*) measured with inverted Dice's score (1 - Dice's score), covariance distance (CovDist), symmetric Hausdorff distance (HD) and normalized symmetric contour distance (NSCD). Only the regions with 1 - Dice's score smaller than 1 have been considered (48 regions out of 89). The very low inverted Dice's scores (or very high Dice's score) proves that few manual interventions were required to obtain a visually optimal results, and that consecutive manual interventions on the same subject were performed consistently.

In this experiment, the rater did not consider necessarily to adjust every region: the macro regions 1, 2, 4, 6, 8, 11 and 12 required no manual intervention after the automatic segmentation. Fig. 9 shows the differences measured with the selected metrics for all the regions that required manual intervention, comparing the automatic segmentation (*auto*) and the two manual adjustments (*adj1* and *adj2*) in three different boxplot.

### Automatic segmentation assessment via cross validation

In a leave-one-out cross validation (Kohavi et al., 1995), each subject underwent again automatic segmentation based on the remaining 11 atlases. For each subject, the obtained automatic segmentation was then compared with the available manual one.

**Fig. 10:**
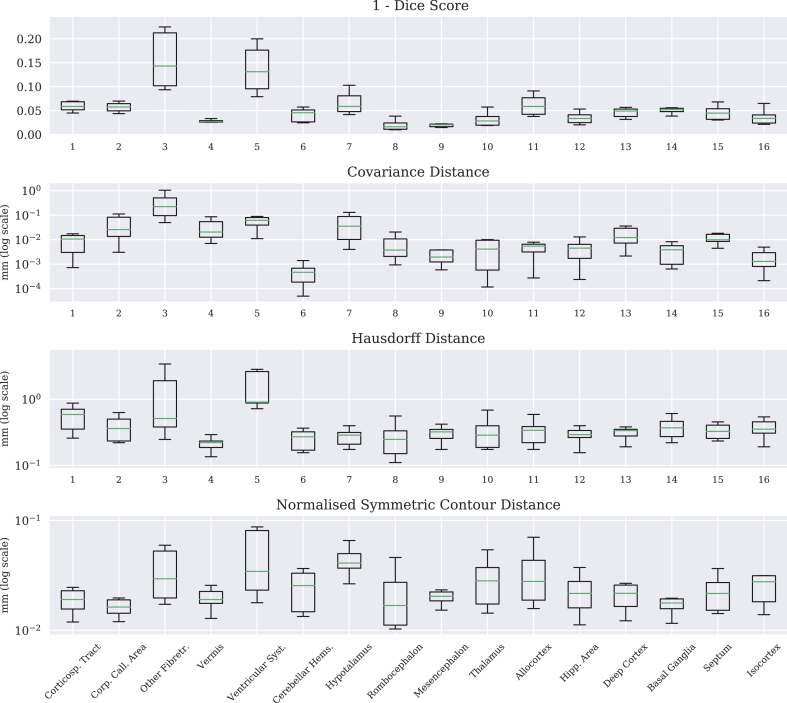
**Leave one out cross validation for the selected macro-regions.** Each point in the boxplot corresponds to the error at the given macro-region and for the given scoring system between the manual ground truth and the propagation of one of remaining 11 subjects. Differences between segmentations are scored with 1 - Dice score, covariance distance, Hausdorff distance and normalized symmetric contour distance, and the last three measures are plotted in log-scale. The correspondence between the x-axis and the macro-regions labels is reported in Table 2.

To simplify the representation, the delineated regions were grouped into a set of 16 macro-regions, proposed in Table 2. The difference between the manual adjustment and the segmentation obtained with the remaining 11 subjects were scored with the four proposed metrics. Results are shown in the box plots in Fig. 10 for the macro-regions.

**Table 2:** **Macro-regions grouping.** For validation purposes, the 89 regions were grouped in to 16 macro-regions. The proposed grouping was constructed by keeping regions of similar importance or anatomy or quality together, in this order of preference.

Label	Region	Grouped sub-labels
1	Corticospinal Tract	223, 224, 225, 226,
		227, 228, 229, 230
2	Corpus Call. Area	218, 219, 220
3	Other Fibretracts	215, 233,237, 239,
		240, 241, 242, 243,
		244, 247,248, 251,
		252, 253
4	Cerebellar Vermis	161
5	Ventricular System	201, 203, 204, 211, 212
6	Cerebellar Hems.	179, 180
7	Hypothalamus	109, 110, 121
8	Rombocephalon	151, 153
9	Mesencephalon	127, 129, 130,133,
		134, 135, 136, 139,
		140, 141, 142
10	Thalamus	83, 84
11	Allocortex	25, 26, 27, 28
12	Hippocampal Area	31, 32, 43,
		44, 45, 46
13	Deep Cortex	53, 54, 55, 56
14	Basal Ganglia	69, 70, 71,
		72, 75, 76
15	Septum, Basal Forebrain	77, 78
16	Isocortex	5, 6, 7, 8, 9, 10, 11,
		12, 13, 14, 15,16,
		17, 18, 19, 20

**Table 3:** Quantification of the inter-rater variability and automatic method in the segmentation of left hippocampus. ${\text{Rater}}_{1}$ (expert) and ${\text{rater}}_{2}$ (non expert) manually segmented the left hippocampus of an unlabeled and randomly selected subject. Resulting segmentations are compared with the automatic one. Differences are assessed with Dice's score, covariance distance (CovDist), symmetric Hausdorff distance (HD) and normalized symmetric contour distance (NSCD).

	Dice	CovDist	HD	NSCD
${\text{rater}}_{1}$ vs ${\text{rater}}_{2}$	0.75	0.301	0.865	0.183
${\text{rater}}_{1}$ vs auto	0.94	0.004	0.441	0.041
${\text{rater}}_{2}$ vs auto	0.73	0.238	0.991	0.214

The boxes show the interquartile interval, whiskers the minimum and the maximum. The central bar shows the median. Macro regions that were better aligned, according to all the selected metrics, were regions 1, 2, 4, 6, 8, 11 and 12.

## Further in vivo experiments

To provide an assessment of the quality of the proposed automatic method and to validate the feasibility on a different dataset further *in vivo* acquisitions were planned. Four neonatal rabbits underwent high resolution MRI with the following protocol:

▷ Structural T1 weighted image acquisition: MRI was performed on living animals under isoflurane anaesthesia using a Bruker Biospec 9.4 T small animal MR scanner (Bruker Biospin, Ettlingen, Germany; horizontal bore, 20 cm) equipped with actively shielded gradients (600 mT/m) utilising a rat brain surface receiver decoupled to a volume quadrature transmit coil (internal diameter of 72 mm). Field homogeneity was corrected using fieldmap correction (MAPSHIM protocol, Bruker Paravision 5.1). Anatomical reference was obtained through a 3D T2 weighted RARE sequences (TR/TE: 42/1000 ms; RARE factor: 8; FOV: 24×30×30 mm; matrix for the first subject 128×128×128. matrix for the remaining subjects: 160×192×192; acquisition time 19 min).
▷ Diffusion-weighted image acquisition: Diffusion-weighted images (DWI) were acquired using a spin-echo version of the echo-planar imaging (SE-EPI) sequence with 4 segments; TE/TR: 27/5000 ms; 2 averages; FOV: 30×30 mm; matrix first subject: 128×128; matrix remaining subjects: 192×192; 20 axial slices of 1 mm thickness with a 0.2 mm gap; 6 directions and 4 B values (100, 500, 1000, 1500 s/mm 2); acquisition time: 16 min.

As the resolution of the DWI resulted too low compared to the T1, it was not possible to obtain satisfactory results with the multi-modal approach described for the *ex vivo* example. The propagation was therefore based only on the T1 modalities. Moreover, the non-rigid bending energy parameter was increased from 0.5 of the *ex vivo* setting to 0.8 for the *in vivo*. The low resolution of the DWI made also difficult to quantify the quality of the segmentation alongside the borders that are visible only in the DWI-based modalities (FA, MD and V1).

**Fig. 11:**
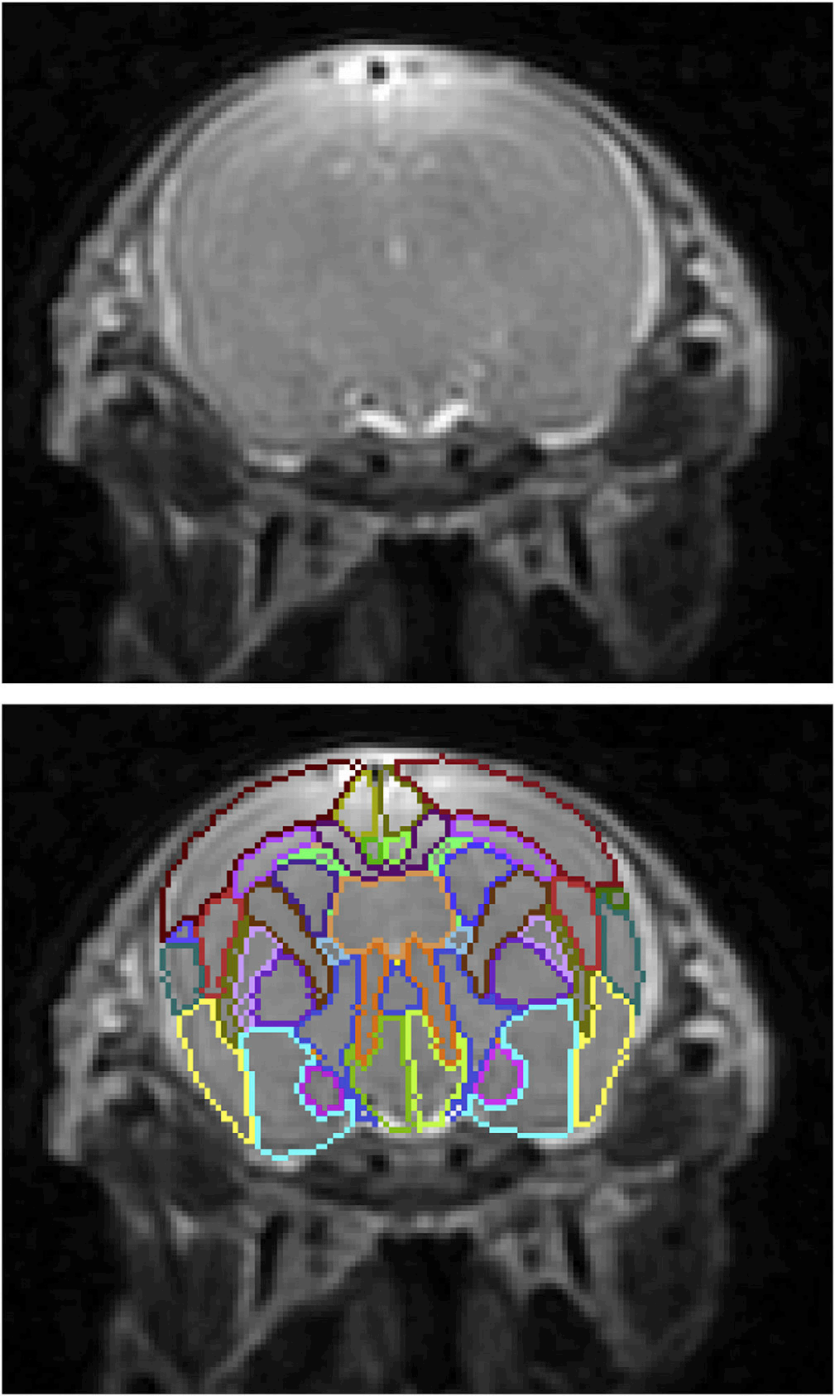
**In vivo experiment.** The result of the automatic algorithm tested on an *in vivo* acquisition. The subject, left in the original orientation, is provided with a visually good segmentation. In this setting, the multi-modal approach was not feasible due to the too low resolution of the diffusion weighted image.

Results are shown in coronal section in Fig. 11 for one subject. Segmentation of the other subjects can be found in the supplementary material D. As no manual segmentation is available for this dataset, this experiment does not provide a numerical validation of the proposed method. Nonetheless, these preliminary segmentation may provide a valuable initialization.

## Discussion

The proposed high-resolution MRI-based multi-atlas provides a tool to create an automatic parcellation for neurological research using MRI imaging of newborn rabbits. It may be beneficial for the translational research focusing on perinatal brain injury and on MRI-based pre-clinical trials.

### Acquisition quality

Acquisition quality is a major determining factor in creating a reference atlas. Herein we opted for an *in skull* acquisition, to avoid an overall shape deformation found in *ex skull* protocols due to extraction artefacts and the long time of the brain in the scanner.

Another benefit could entail the better segmentation propagation when translating the multi atlas on an *in vivo* acquisition of future studies. Preliminary results in this direction, shown in Fig. 11 and suppl. D, show that the proposed method adapts well when a different protocol is employed. The proposed method could significantly reduce the manual intervention for the creation of a future *in vivo* multi-atlas.

### Artefacts

Susceptibility artefacts have had an impact on the design of the propagation algorithm as well. These artefacts, caused by local magnetic field inhomogeneities resulted in unwanted misalignment in the non-rigid registration processing phase. Furthermore the presence of artefacts especially affecting in the DWI-based modalities was problematic.

Susceptibility artefacts were most likely a consequence of the presence of air bubbles in the gadoteridol-paraformaldehyde composition, which can accumulate between the brain and skull during the perfusion. An improvement in image quality was observed during the study, when applying degasification process to the gadoteridol-paraformaldehyde composition prior to the perfusion. Further studies should therefore consider the inclusion of this step in the sample preparation.

### Validation

The customary Dice score alone is not enough to provide a numerical representation of the differences between two shapes. The four selected metrics, other than quantifying the overlap, provide the overall shape direction distribution, the upper bound and the average discrepancy between borders.

In the inter-rater variability test-retest, the expert rater (${\text{rater}}_{1}$, Table 3) appears to be very close to the automatic method, with a maximal distance between the two regions border equal to 0.441 mm, and an average distance equal to 0.041 mm. A higher variability can be noticed between the expert and non expert rater, despite following the same manual segmentation protocol. On one hand this result is biased by the fact that ${\text{rater}}_{1}$, is the same that created the Multi-Atlas that provided the automatic method. On the other hand, this outcome provides a confirmation of the inter rater-variability, even if following the same protocol, emphasising the importance of an automatic method, or an automatic initialization.

The strategy of iterating three times between automatic method and manual adjustments led to a continuous improvement, as proved in the anonymized segmentation visual scoring (Subsection 4.3).

The outcome of the leave one out cross validation for the 12 subjects shown in Fig. 10, provides a result coherent with the outcome of the intra-rater test-retest experiment. Regions 1, 2, 4, 6, 8, 11 and 12 that obtained the highest dice score are the regions that required no manual intervention. In supplementary material E the boxplot is overlayed with the outcome of the two manual adjustments of the intra-rater test-retest.

The macro region with the worst alignment was *other Fibretracts*. This region includes most of the elongated anatomies (such as Optic tracts and anterior commissure), whose misalignment is better captured by the covariance distance. If measured with the Hausdoroff distance, the same region performed better than the *ventricular system*, that is the most difficult to delineate manually, in particular in the region between the skull and the retrosplenium left and right.

The normalized symmetric contour distance can be seen as robust to outliers Hausdoroff distance. If for example two regions are perfectly aligned apart from one column of voxels protruding in radial direction from one of the regions, their Hausdoroff distance equals the length of the protruding line of voxels. The normalized symmetric contour distance in the same situation is almost zero, not being influenced by this single roughness. Comparing the results of these two measurements in the leave-one-out experiment, the Hypotalamus results to be the region less affected by outliers respect to the other regions.

In general, the high scores in the leave-one-out highlight a stability in the regions propagation. Moreover the higher Dice score and the consistency in the intra-rater manual adjustment variability shows that the outcome of the automatic algorithm is qualitatively high, requiring small amount of manual adjustment.

### Reliability

The key question relates to the representativeness and the consequent transferability of the proposed multi-atlas on a different dataset. The uneven distribution between term and pre-term and among male and female subjects (Fig. 1) may bias the propagation over a subject that is under-represented in the population. Nevertheless, the distribution of brain and body weights seems to be wide enough to capture a likewise wide biological variability.

The *in vivo* experiments showed that even if the proposed algorithm is applied over an image acquired with an different protocol, most of the regions were subjectively considered well aligned.

On the algorithmic side, for the fine-tuning parameters we mostly relied on visual assessment. As the manual segmentation quality went through a process of gradual improvement throughout the study, a ground truth had not been available to apply a more sophisticated numerical method. Grid-based or random search methods may, if applied in future studies, contribute to improve the propagation results over a wider range of acquisitions.

## Conclusion

In this paper we have proposed the first new-born rabbit brain multi-atlas, acquired at 9.4 T and segmented with a semi-automatic method using the T1 weighted images and a range of DWI modalities.

Due to the differences between rat and rabbit brain anatomy, we proposed a stereotaxic orientation based on readily identifiable landmarks and compatible with longitudinal studies. This is also aimed at facilitating any future comparison or study involving histology and MRI in the newborn rabbit and longitudinal studies.

Subsequent studies, which may potentially benefit from the work presented here, might require their own specific refined segmentation, parameter tuning and taxonomy. For this reason, in analogy with collaborative software development, the segmentations are proposed in a versioned controlled open repository (https://github.com/gift-surg/SPOT-A-NeonatalRabbit). Each further improvement and each different taxonomical subdivision can be uploaded, while keeping track of the previous multi-atlas version with a unique identifier, to guarantee reproducibility.
