# Supplementary material for: A magnetic resonance multi-atlas for the neonatal rabbit brain
Source: Neuroimage. 2018 Oct 1;179:187–98. doi: 10.1016/j.neuroimage.2018.06.029 (PMC6203700; doi:10.1016/j.neuroimage.2018.06.029)
Supplement: supplementary_material_A [file mmc1.pdf]

Appendix A: Bicommissural and histologically-compatible stereotaxic orientation

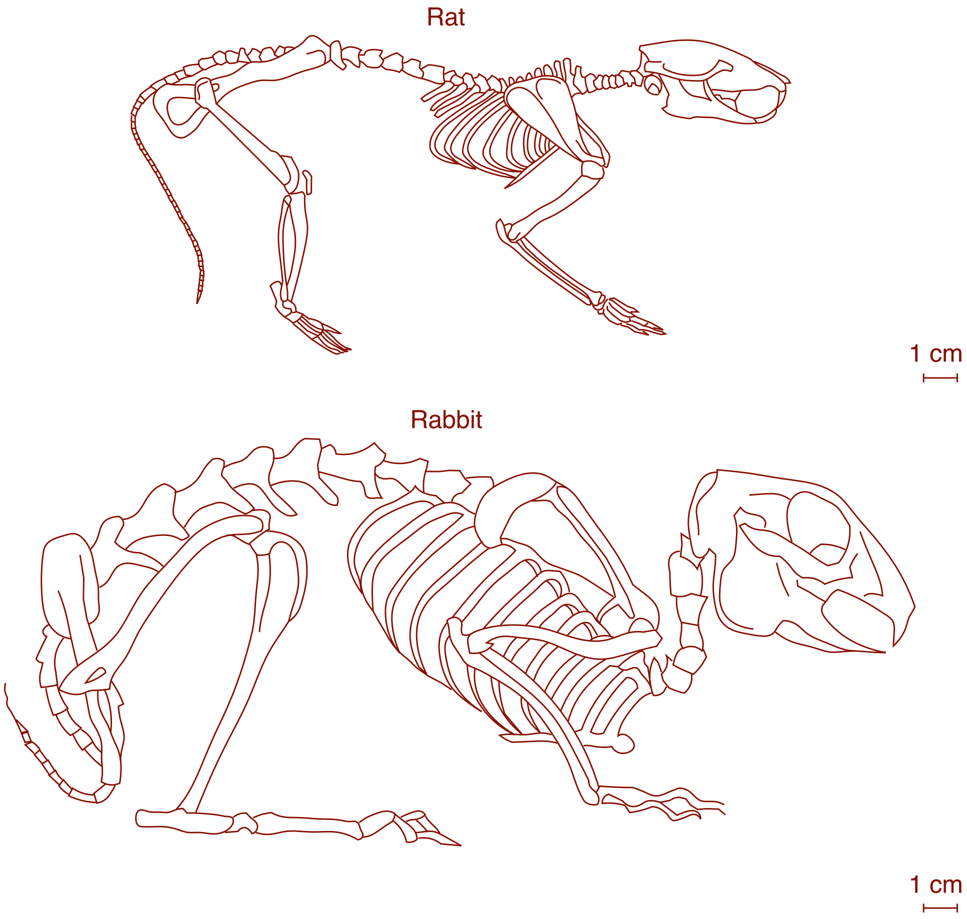

Figure 1: **Comparison between adult rat and rabbit skeletons.** Comparison between the adult rat and rabbit skeletons. Note the difference in latent posture and head position resulting in skull attitude differentiation. Inspired by https://www.behance.net/gallery/12930617/Rabbit-skeleton and http://bcrc.bio.umass.edu/intro/content/rat-dissection-protocol. Last access June 6, 2018.

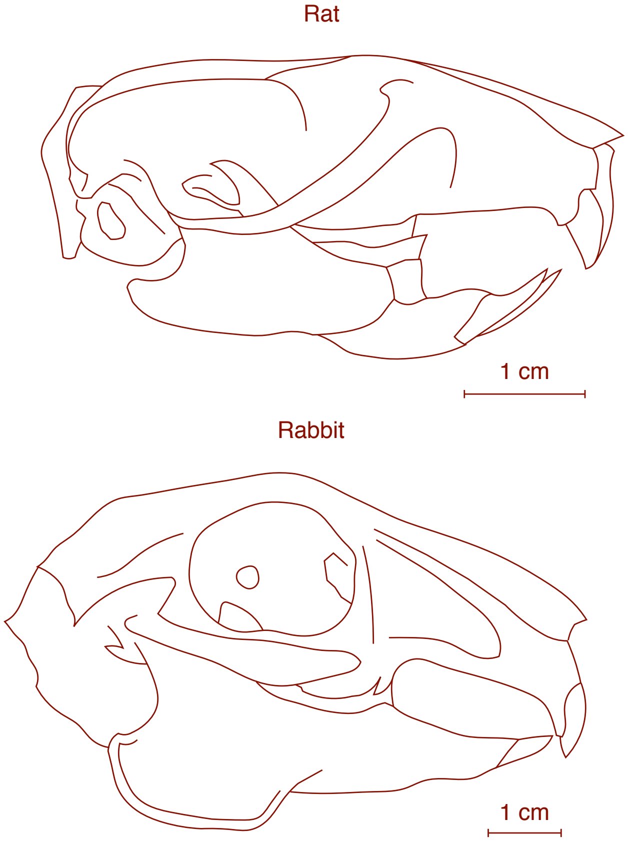

Figure 2: **Comparison between adult rat and rabbit skulls.** Comparison between the adult rat and rabbit skull. Note the shape difference of the frontal and parietal skull shape. Inspired by http://www.skullsite.co.uk/Lagomorphs/lagomorphs.htm and http://www.skullsite.co.uk/Rat/comrat.htm.

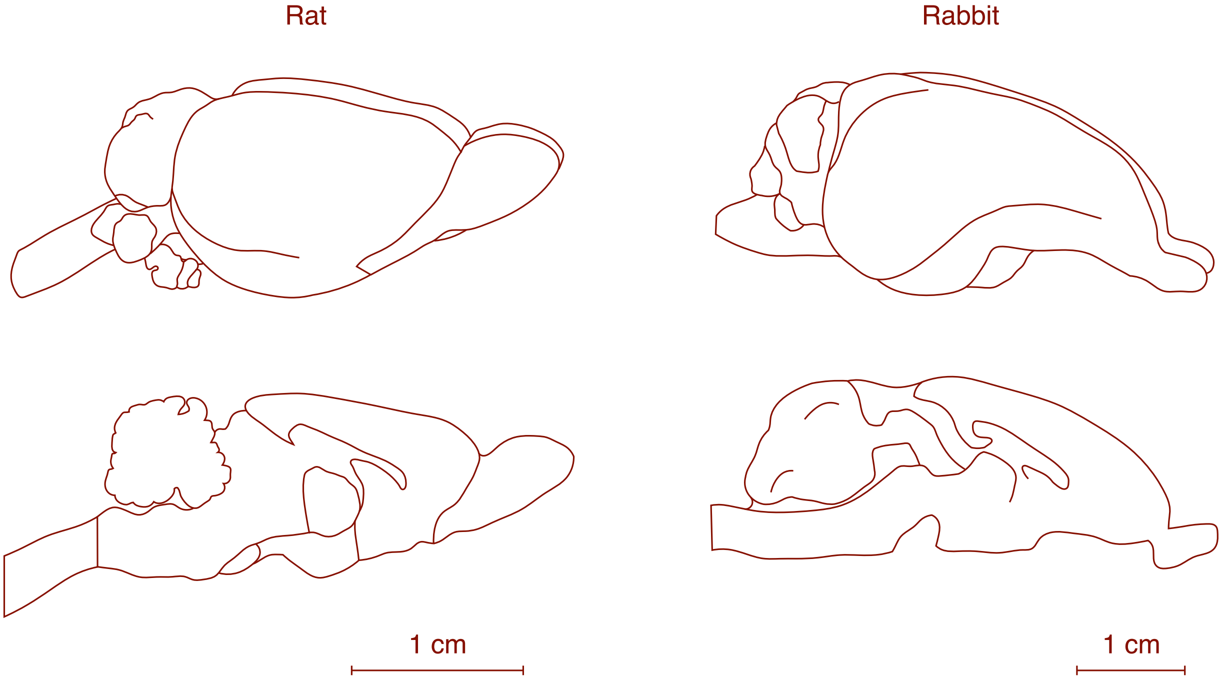

Figure 3: **Rat and rabbit brain compared.** Representation of the newborn rat and rabbit brain. 3D representative sketches (top) and mid-sagittal section (bottom). Inspired by http://www.medicinafetalbarcelona.org/rabbitbrainatlas/ and http://www.skullsite.co.uk/Rat/comrat.htm.

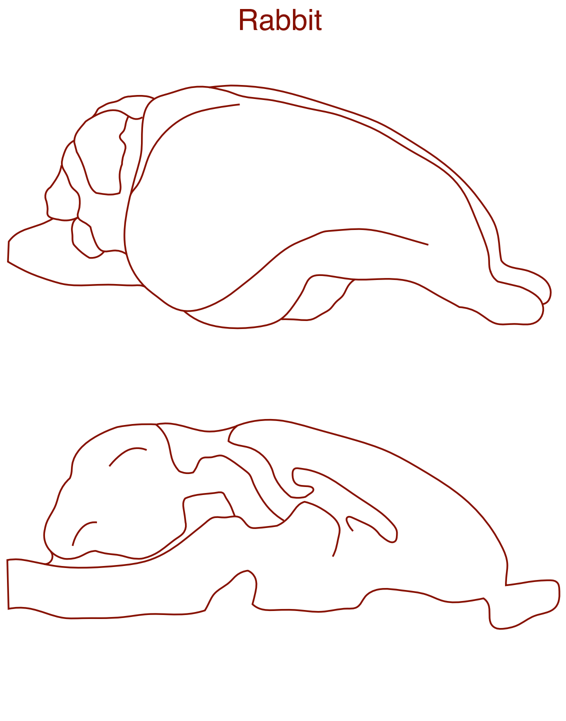

Figure 4: **Comparison between newborn and adult rabbit brain.** Comparison between newborn (right) and adult (left) rabbit brain. with 3D representative sketches (top) and midsagittal section (bottom). Inspired by http://www.medicinafetalbarcelona.org/rabbitbrainatlas/ and the proposed multi-atlas https://github.com/gift-surg/SPOT-A-NeonatalRabbit.

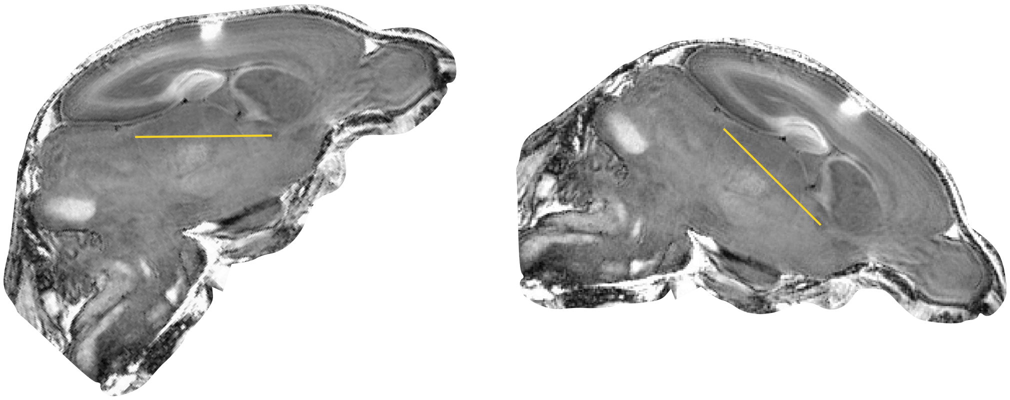

(a)

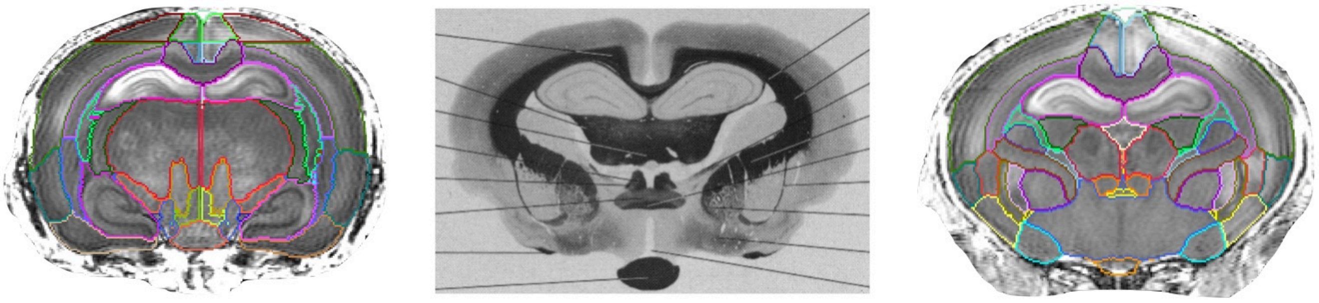

(b)

Figure 5: **Bicommissural versus histologically-compatible orientation. (a)** Left: MRI T1 sagittal section in bicommissural orientation, customary for rats does not produce coronal sections compatible with the available histological atlases. Right: the proposed stereotaxic orientation (bicommissural plane forms a $45°$ angle with the horizontal plane). **(b)** Comparison between the coronal sections obtained in bicommissural orientation (left), the adult histological atlas (Centre) [Shek, J. W., Wen, G. Y., Wisniewski, H. M., 1986. Atlas of the rabbit brain and spinal cord. S Karger Pub.] and the coronal sections in the proposed orientation (Right).
