# Supplementary material for: A magnetic resonance multi-atlas for the neonatal rabbit brain
Source: Neuroimage. 2018 Oct 1;179:187–98. doi: 10.1016/j.neuroimage.2018.06.029 (PMC6203700; doi:10.1016/j.neuroimage.2018.06.029)
Supplement: supplementary_material_B [file mmc2.pdf]

Appendix B: Proposed taxonomy for the newborn brain rabbit

Hierarchy	region	Abbreviation	Number
0	Brain [encephalon]	BR	
1	Cerebrum [cerebral hemispheres, endbrain, telencephalon]	CH / Tel	1
1.1	Cerebral cortex	Cx	2
1.1.1	Cortical Plate	Cxpl	3
1.1.1.1	Isocortex/neocortex		4
1.1.1.1.1	Prefrontal area	PFrA	5, 6
1.1.1.1.2	Frontal area	FrA	7, 8
1.1.1.1.3	Occipital area	OA	9, 10
1.1.1.1.4	Parietal area	PtA	11, 12
1.1.1.1.5	Temporal area	TeA	13, 14
1.1.1.1.6	Cingulate area	Cg	15, 16
1.1.1.1.7	Retrosplenial area	RS	17, 18
1.1.1.1.8	Insular area	Ins	19, 20
1.1.1.1.9	Ectorhinal area	Ect	21, 22
1.1.1.2	Allocortex		23
1.1.1.2.1.1	Olfactory lobe	OB	25, 26
1.1.1.2.1.2	Piriform	Pir	27, 28
1.1.1.3	Hippocampal formation	HF	29
1.1.1.3.1	Hippocampal area	HA	31, 32
1.1.1.3.1.1	Ammons horn CA1 area	CA1	33, 34
1.1.1.3.1.2	Ammons horn CA2 area	CA2	35, 36
1.1.1.3.1.3	Ammons horn CA3 area	CA2	37, 38
1.1.1.3.1.4	Dentate Gyrus	DG	39, 40
1.1.1.3.2	Parahippocampal area	PHA	41, 42
1.1.1.3.2.1	Subiculum	S	43, 44
1.1.1.3.2.2	Entorhinal area	Ent	45, 46
1.1.1.3.2.3	Perirhinal area	PRh	47, 48
1.1.2	Cortical subplate, deep cortex	Cxsp	51
1.1.2.1	Claustrum	Cl	53, 54
1.1.2.2	Amygdala	Am	55, 56
1.2	Cerebral nuclei / basal ganglia	BG	65
1.2.1	Striatum	Str	67, 68
1.2.1.1	Caudate nucleus	CA CN	69, 70
1.2.1.2	Putamen	Pu	71, 72
1.2.2	Pallidum	Pa	73, 74
1.2.2.1	Globus Pallidus	GP	75, 76
1.2.3	Basal forebrain	BF	77
1.2.4	Septum Area	SA	78
2	Brainstem		80
2.1	Interbrain [diencephalon]	IB / D	81
2.1.1	Thalamus	THA	83, 84
2.1.1.1	Epithalamus	EPI	85, 86
2.1.1.1.1	Pineal body	Pi	87, 88

2.1.1.1	Dorsal thalamus	DOR	89, 90
2.1.1.1.1	Anterior thalamic nuclei	AD	91, 92
2.1.1.1.2	Geniculate nuclei	GEN	93, 94
2.1.1.1.3	Intralaminar nuclei	ILM	95, 96
2.1.1.1.4	Lateral thalamic nuclei	LAT	97, 98
2.1.1.1.5	Medial thalamic nuclei	AM	99, 100
2.1.1.1.6	Midline thalamic nuclei	MID/MTN	101, 102
2.1.1.1.7	Ventral thalamic nuclei	VENT	103, 104
2.1.1.2	Ventral thalamus	VNT (VT)	105, 106
2.1.1.2.1	(Ventral lateral) Geniculate nuclei	LGv /GEN	107, 108
2.1.1.2.2	Reticular nuclei	RT	
2.1.2	Hypothalamus	HYP	109, 110
2.1.2.1	Periventricular zone	PVZ	111, 112
2.1.2.2	medial zone	MEZ	113, 114
2.1.2.2.1	Preoptic level	PRO	115, 116
2.1.2.2.2	Anterior level	ANT	117, 118
2.1.2.2.3	Tuberal level	TUB	119, 120
2.1.2.2.4	Mammillary body	MAM	121
2.1.2.3	Lateral zone	LZ	123, 124
2.2	Midbrain [mesencephalon]	MB	127
2.2.1	Pretectal region	PRT	129, 130
2.2.2	Tectum	TC	131, 132
2.2.2.1	Superior colliculus	SC	133, 134
2.2.2.2	Inferior colliculus	IC	135, 136
2.2.3	Tegmentum	TG	137, 138
2.2.3.1	Substantia nigra	SN	139, 140
2.2.3.2	Periaqueductal gray	PAG	141, 142
2.2.3.3	Ventral tegmental area	VTA	143, 144
2.2.3.4	Red nucleus	RN	145, 146
2.2.3.5	Reticular formation		147, 148
2.3	Hindbrain [rhombencephalon]	HB	149
2.3.1	Pons [metencephalon]	PO	151
2.3.2	Medulla [myelencephalon]	MY	153
3	Cerebellum (parencephalon)	Cb	158
3.1	Cerebellar cortex	Cbx	159, 160
3.1.1	Vermal Regions	VERM	161
3.1.1.1	Lingula	LING	163, 164
3.1.1.2	Central lobe	CENT	165, 166
3.1.1.3	Culmen	CUL	167, 168
3.1.1.4	Declive	DEC	169, 170
3.1.1.5	Folium-tuber vermis	FOTU	171, 172
3.1.1.6	Pyramus	PYR	173, 174
3.1.1.7	Uvula	UVU	175, 176
3.1.1.8	Nodulus	NOD	177, 178
3.1.2	Hemispheric Regions	HEM	179, 180
3.1.2.1	Simple lobe	SIM	181, 182
3.1.2.2	Ansiform lobe	AN	183, 184
3.1.1.1	Paramedian lobe	PRM	185, 186
3.1.1.2	Copula pyramidis	COPY	187, 188
3.1.1.3	Paraflocculus	PFL	189, 190
3.1.1.4	Flocculus	FL	191, 192
3.2	Cerebellar nuclei	CBN	193, 194

3.2.1.1	Fastigial nucleus	FN	195, 196
3.2.1.2	Interposed nucleus	IP	197, 198
3.2.1.3	Dentate nucleus	DN	199, 200
4	Ventricular system	VS	201
4.1	Interventricular foramen	IVF	202
4.2	Lateral ventricle	LV	203, 204
4.3	Third ventricle	3V	205
4.4	Cerebral aqueduct	Aq	206
4.5	Fourth ventricle	4V	207
4.6	Central canal	CC	208
4.7	Choroid plexus	chp	209
4.8	Velum interpositum / superior medullary velum	VIP/SMV	210
4.9	Periventricular area	PV	211, 212
5	Fiber tracts:		213
5.1	Cranial nerves		214
5.1.1	Optic tract and optic chiasm	OT	215
5.1.2	Lateral olfactory tract	lo	216
5.2	Lateral forebrain bundle system		217
5.2.1	Corpus callosum	cc	218
5.2.2	External capsule	ec	219, 220
5.2.3	Corticospinal tract		221, 222
5.2.3.1	Internal capsule	int	223,224
5.2.3.2	Corona radiata	cr	225, 226
5.2.3.3	Cerebal peduncle	cp	227, 228
5.2.3.4	Subcortical white matter	swm	229, 230
5.3	Medial forebrain bundle system		231
5.3.1	Anterior commissure	ac	233
5.3.2	Fornix system	fxs	235, 236
5.3.2.1	Hippocampal commissure	hc	237
5.3.2.2	Fimbria of hippocampus	fi	239, 240
5.3.2.3	Columns of the fornix	fx	241, 242
5.3.3	Stria terminals	st	243,244
5.4	Hypothalamus related		245
5.4.1	Mammilothalmic tract	mt	247,248
5.4.2	Stria medullaris	sm	249, 250
5.4.3	Fasciculus tetroflexus	fr	251,252
5.4.4	Posterior commissure	pc	253
