# Supplementary material for: A magnetic resonance multi-atlas for the neonatal rabbit brain
Source: Neuroimage. 2018 Oct 1;179:187–98. doi: 10.1016/j.neuroimage.2018.06.029 (PMC6203700; doi:10.1016/j.neuroimage.2018.06.029)
Supplement: supplementary_material_C [file mmc3.pdf]

Appendix C: Manual Delineation Protocol

	Regions	Abbrev	Notes
1	Cerebral Cortex	Cx	
1.1	Prefrontal Area	PFrA	Structures 5 and 6: medial frontal cortex (right; left)
	Orientation of slices		Coronal
	Anterior border		Superior → inferior: frontal cortex, olfactory bulb
	Posterior border		Superior → inferior: cingulate cortex
	Medial border		Superior → inferior: CSF, none
	Lateral border		Superior → inferior: CSF, frontal cortex, corpus callosum
	Superior border		Frontal cortex, CSF
	Inferior border		Frontal cortex, basal forebrain, corpus callosum
1.2	Frontal area	FrA	Structures 7 and 8: frontal cortex (right; left)
	Orientation of slices		Coronal
	Anterior border		Superior → inferior: CSF, olfactory bulb
	Posterior border		Superior → inferior: parietal cortex, temporal cortex
	Medial border		Superior → inferior: CSF, medial frontal cortex, cingulate cortex, corona radiata
	Lateral border		CSF
	Superior border		CSF
	Inferior border		Insular, temporal cortex, corona radiata
1.3	Occipital area	OA	Structures 9 and 10: occipital cortex (right; left)
	Orientation of slices		Coronal
	Anterior border		First slice: parietal cortex, first slice in which hippocampi meet laterally
	Posterior border		Last slice: entorinal cortex and CSF
	Medial border		CSF
	Lateral border		Anterior → posterior: parietal cortex, temporal cortex, entorinal cortex, CSF
	Superior border		CSF
	Inferior border		Anterior → posterior: corona radiate, subcortical white matter, retrosplenium
1.4	Parietal area	PtA	Structures 11 and 12: parietal cortex (right; left)
	Orientation of slices		Coronal
	Anterior border		First slice: most anterior slice in which hippocampi appear, frontal cortex
	Posterior border		Last slice: slice in which hippocampi meet laterally, occipital cortex
	Medial border		Anterior → posterior: corpus callosum, corona radiate, subcortical white matter
	Lateral border		Anterior → posterior: CSF, temporal cortex
	Superior border		Anterior → posterior: CSF, retrosplenium
	Inferior border		Anterior → posterior: claustrum, temporal cortex, subcortical white matter
1.5	Temporal area	TeA	Structures 13 and 14: temporal cortex (right; left)
	Orientation of slices		Coronal
	Anterior border		First slice: slice in which hippocampi appear, insular cortex
	Posterior border		Last slice: entorhinal cortex, subcortical white matter, occipital cortex
	Medial border		Anterior → posterior: claustrum, corona radiata, subcortical white matter, CSF
	Lateral border		Anterior → posterior: CSF
	Superior border		Anterior → posterior: parietal cortex, occipital cortex, CSF
	Inferior border		Anterior → posterior: piriform cortex, entorhinal cortex, CSF

1.6	Cingulate area	Cg	Structures 15 and 16: cingulate cortex (right; left)
	Orientation of slice		Coronal
	Anterior border		First slice: most anterior slice in which the genu of the corpus callosum is visualized, medial prefrontal cortex
	Posterior border		Last slice: slice anterior to that in which hippocampus appears (as previously defined) , retrosplenium
	Medial border		Anterior → posterior: no medial border
	Lateral border		Anterior → posterior: frontal cortex, subcortical white matter
	Superior border		Anterior → posterior: frontal cortex, CSF
	Inferior border		Anterior → posterior: corpus callosum
1.7	Retrosplenial area	RS	Structures 17 and 18: retrosplenium (right; left)
	Orientation of slices		Coronal
	Anterior border		First slice: anterior to that in which hippocampus appears, cingulate cortex
	Posterior border		midbrain, CSF
	Medial border		Anterior → posterior: no medial border , CSF
	Lateral border		Anterior → posterior: parietal cortex, occipital cortex, subcortical white matter
	Superior border		Anterior → posterior: parietal and occipital cortex, CSF
	Inferior border		Anterior → posterior: corpus callosum, subiculum, midbrain
1.8	Insular area	Ins	Structures 19 and 20: insular area (right; left)
	Orientation of slices		Coronal
	Anterior border		First slice: where periventricular area appears, frontal cortex
	Posterior border		temporal cortex, CSF
	Medial border		Anterior → posterior: claustrum, subcortical white matter
	Lateral border		CSF
	Superior border		Anterior → posterior: frontal cortex
	Inferior border		Anterior → posterior: piriform cortex
1.9	Entorhinal area	Ent	Structures 45 and 46: entorhinal area (right; left)
	Orientation of slices		Coronal
	Anterior border		First slice where piriform cortex appears
	Posterior border		CSF
	Medial border		Anterior → posterior: subcortical white matter, hippocampus and subiculum
	Lateral border		CSF
	Superior border		Anterior → posterior: temporal cortex, occipital cortex
	Inferior border		Anterior → posterior: piriform cortex, amygdala, CSF
1.10	Olfactory lobe	OB	Structures 25 and 26: olfactory (right; left)
	Orientation of slices		Coronal
	Anterior border		CSF
	Posterior border		basal midbrain, periventricular area
	Medial border		Anterior → posterior: basal forebrain, periventricular area and frontal cortex
	Lateral border		CSF
	Superior border		Anterior → posterior: upper-limited by rhinal fissure, frontal cortex
	Inferior border		CSF
1.11	Piriform	Pir	Structures 27 and 28: piriform (right; left)
	Orientation of slices		Coronal
	Anterior border		First slice: where claustrum area appears, frontal cortex
	Posterior border		Anterior → posterior: amygdala, entorhinal cortex
	Medial border		Anterior → posterior: claustrum, external capsula, amygdala, subcortical white matter
	Lateral border		CSF

	Superior border		Anterior → posterior: insular, temporal cortex
	Inferior border		Anterior → posterior: basal forebrain, amygdala
1.12	Hippocampal area	HA	Structures 31 and 32: hippocampus (right; left)
	Orientation of slices		Coronal
	Anterior border		First slice: slice in which cingulate cortex disappears
	Posterior border		Last slice: formed by subiculum and occipital cortices are
	Medial border		Anterior → posterior: cortical white matter, CSF, thalamus, brain stem
	Lateral border		Anterior → posterior: lateral ventricles, cortical white matter (corona radiate and subcortical white matter), temporal cortex
	Superior border		Anterior → posterior: cortical white matter (corona radiate and subcortical white matter), lateral ventricles
	Inferior border		Anterior → posterior: CSF, thalamus, amygdala, temporal cortex
1.13	Claustrum	Cl	Structures 53 and 54: claustrum (right; left)
	Orientation of slices		Coronal
	Anterior border		First slice: defined on the most anterior slice in which the corona radiata is seen
	Posterior border		Last slice: last slice in which the corona radiata is seen
	Medial border		Anterior → posterior: corona radiata, external capsula
	Lateral border		Anterior → posterior: insular cortex, piriform cortex, temporal cortex
	Superior border		Anterior → posterior: corona radiata, frontal cortex, temporal cortex
	Inferior border		Anterior → posterior: piriform cortex
1.14	Amygdala	Am	Structures 55 and 56: amygdala (right; left)
	Orientation of slices		Coronal
	Anterior border		First slice: defined on the most anterior slice in which the anterior commissure is seen, basal forebrain
	Posterior border		entorhinal cortex
	Medial border		Anterior → posterior: basal forebrain, hippocampus
	Lateral border		Anterior → posterior: piriform cortex, entorhinal cortex
	Superior border		Anterior → posterior: putamen, globus palludus, internal capsula, hippocampus
	Inferior border		CSF
2	Cortical subplate, deep cortex		
2.1	Caudate nucleus	CN	Structures 69 and 70: caudate-putamen (right; left)
	Orientation of slices		Coronal
	Anterior border		First slice: slice where lateral ventricles are clearly visible, periventricular area
	Posterior border		Last slice: slice where corona radiate is visible, lateral ventricle area
	Medial border		Anterior → posterior: periventricular area, septum, lateral ventricles, corona radiata
	Lateral border		Anterior → posterior: putamen, internal capsula
	Superior border		Anterior → posterior: periventricular area, lateral ventricles
	Inferior border		Anterior → posterior: basal forebrain, internal capsula, stria terminalis
2.2	Putamen	Pu	Structures 71 and 72: putamen (right; left)
	Orientation of slices		Coronal
	Anterior border		First slice: slice in which internal capsula are clearly visible, caudate nucleus
	Posterior border		globus pallidus, external capsula
	Medial border		Anterior → posterior: caudate nucleus, internal capsula, globus pallidus
	Lateral border		external capsula
	Superior border		internal capsula
	Inferior border		Anterior → posterior: anterior commissure, basal forebrain
2.3	Globus Pallidus	GP	Structures 75 and 76: caudate-putamen (right; left)
	Orientation of slices		Coronal

	Anterior border		Putamen
	Posterior border		Basal forebrain, putamen
	Medial border		Internal capsula
	Lateral border		Anterior → posterior: putamen, external capsula
	Superior border		Internal capsula
	Inferior border		Anterior → posterior: anterior commissure, basal forebrain, amygdala
3	Brainstem		
3.1	Thalamus	THA	Structures 83 and 84: thalamus (right; left)
	Orientation of slices		Coronal
	Anterior border		First slice: most anterior slice in which columns of the fornix are seen, septum
	Posterior border		Last slice: starting on slice in which the aqueduct of Silvius is distinguished, midbrain
	Medial border		Anterior → posterior: midbrain, brain stem
	Lateral border		Anterior → posterior: stria terminalis, fimbria hippocampus, lateral ventricles, hippocampus
	Superior border		Anterior → posterior: hippocampus, subiculum, pretectal area
	Inferior border		Anterior → posterior: basal forebrain, cerebral peduncle, hypothalamus, midbrain
3.2	Hypothalamus	HYP	Structures 109 and 110: hypothalamus (right; left)
	Orientation of slices		Coronal
	Anterior border		First slice: most anterior slice in which optic chiasm is seen, basal forebrain
	Posterior border		Midbrain, mammillary body
	Lateral border		Anterior → posterior: basal forebrain, columns of fornix, mammothalamic tract
	Superior border		Anterior → posterior: basal forebrain, thalamus, midbrain
	Inferior border		Anterior → posterior: optic tract, basal forebrain, CSF, mammillary body
4	Ventricular system		
4.1	Periventricular area	PV	Structures 211 and 212: lateral ventricle (right; left)
	Orientation of slices		Coronal
	Anterior border		First slice: most anterior slice in which the lateral ventricles are seen, frontal cortex
	Posterior border		lateral ventricular area
	Medial border		Anterior → posterior: corpus callosum
	Lateral border		Anterior → posterior: basal forebrain, frontal cortex, caudate nucleus
	Superior border		Anterior → posterior: corpus callosum, corona radiata
	Inferior border		Anterior → posterior: basal forebrain
4.2	Lateral ventricles	LV	Structures 203 and 204: lateral ventricle (right; left)
	Orientation of slices		Coronal
	Anterior border		First slice: most anterior slice in which the genu of the corpus callosum is seen, periventricular area
	Posterior border		Last slice: slice anterior to where the hippocampus goes laterally
	Medial border		Anterior → posterior: corpus callosum, hippocampus, thalamus
	Lateral border		Anterior → posterior: caudate-nucleus, corona radiata, subcortical white matter
	Superior border		Anterior → posterior: corona radiate, hippocampus
	Inferior border		Anterior → posterior: caudate-nucleus, internal capsula
