# Supplementary material for: A magnetic resonance multi-atlas for the neonatal rabbit brain
Source: Neuroimage. 2018 Oct 1;179:187–98. doi: 10.1016/j.neuroimage.2018.06.029 (PMC6203700; doi:10.1016/j.neuroimage.2018.06.029)
Supplement: supplementary_material_D [file mmc4.pdf]

Appendix D: in vivo preliminary experiments

The proposed automatic segmentation method was applied in a further experiment on an external dataset, acquired with a different protocol. We selected four neonatal rabbits to undergo *in vivo* acquisition. The first subject of the stack was acquired with lower resolution (128 x 128 x 128) as an initial test. Remaining subjects were acquired at an higher resolution (160 x 192 x 192). The protocol is provided in the paper, section 5.

The results shown below are computed only on the T1 modality with an increase in the bending energy for the non-rigid registration step (from 0.5 ex vivo to 0.8 in vivo). With the selected protocol the resolution of the DWI was too low to successfully use the multi modal automatic segmentation algorithm as proposed in the manuscript. Only representative sections of the four subjects with the automatic segmentations, with no manual refinement, are shown below.

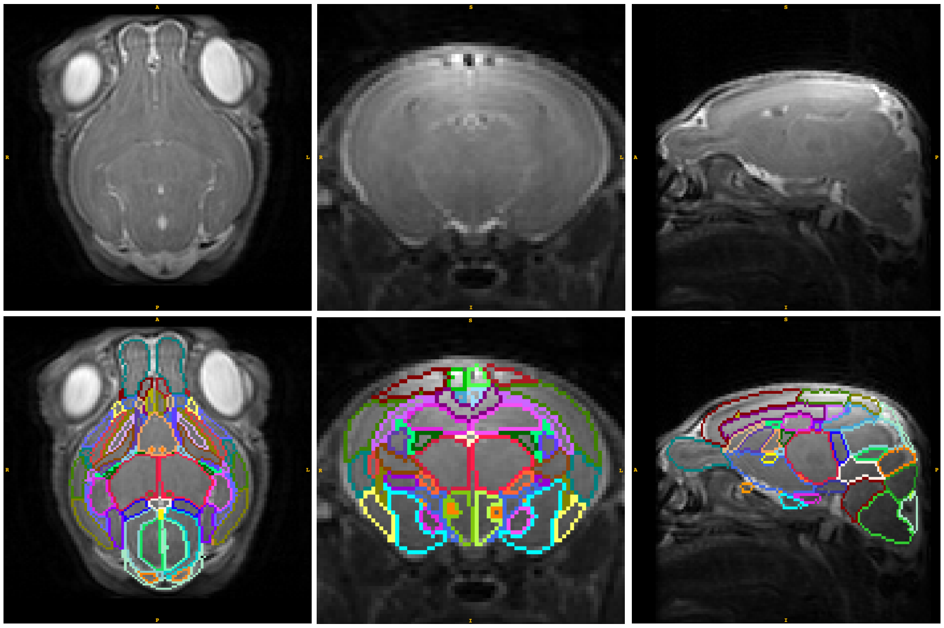

Figure 1: **Visual assessment for the in vivo automatic segmentation method.** First subject, resolution 128 x 128 x 128.

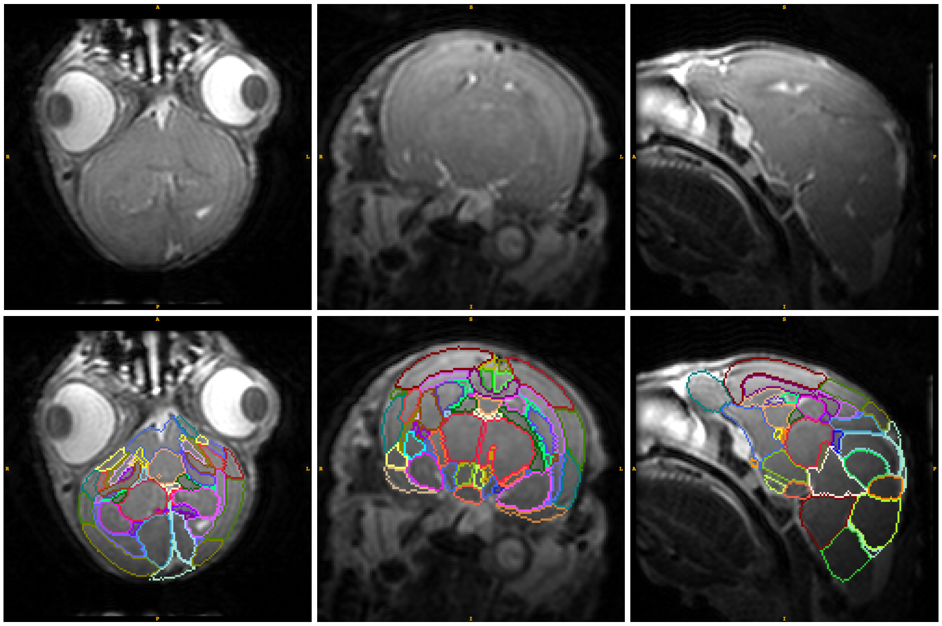

Figure 2: **Visual assessment for the in vivo automatic segmentation method.** Second subject, resolution 160 x 192 x 192.

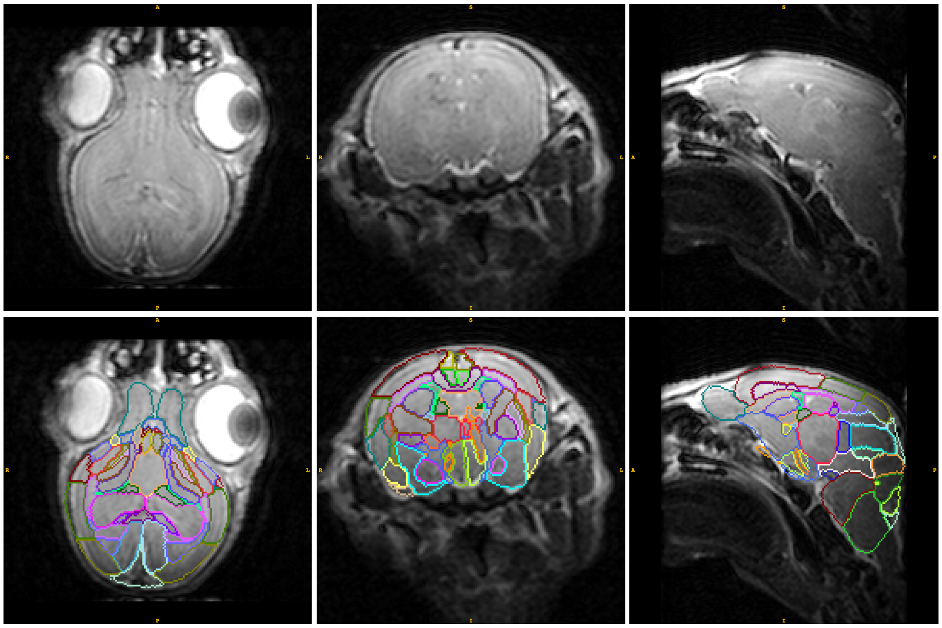

Figure 3: **Visual assessment for the in vivo automatic segmentation method.** Third subject, resolution 160 x 192 x 192.

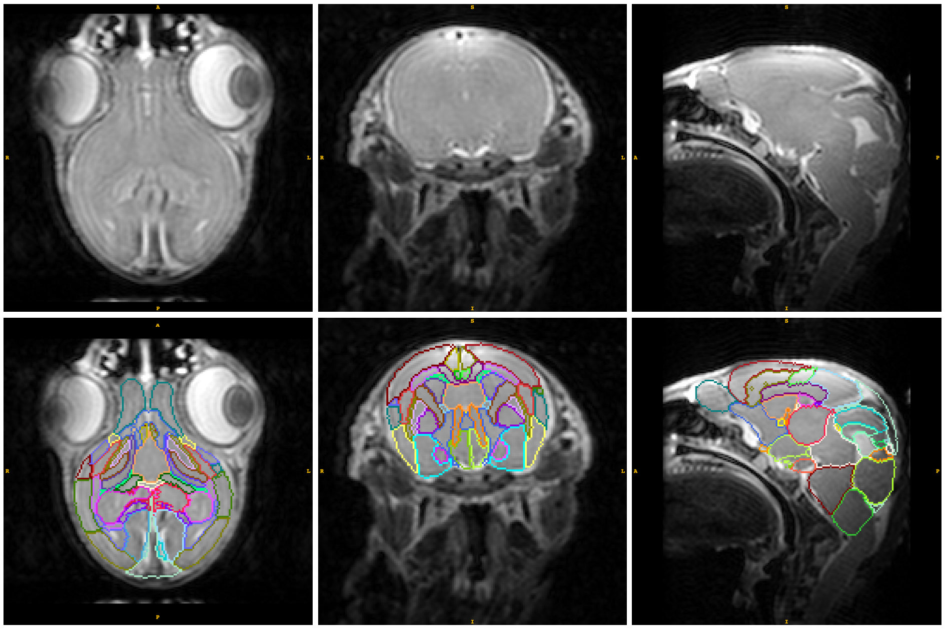

Figure 4: **Visual assessment for the in vivo automatic segmentation method.** Fourth subject, resolution 160 x 192 x 192.

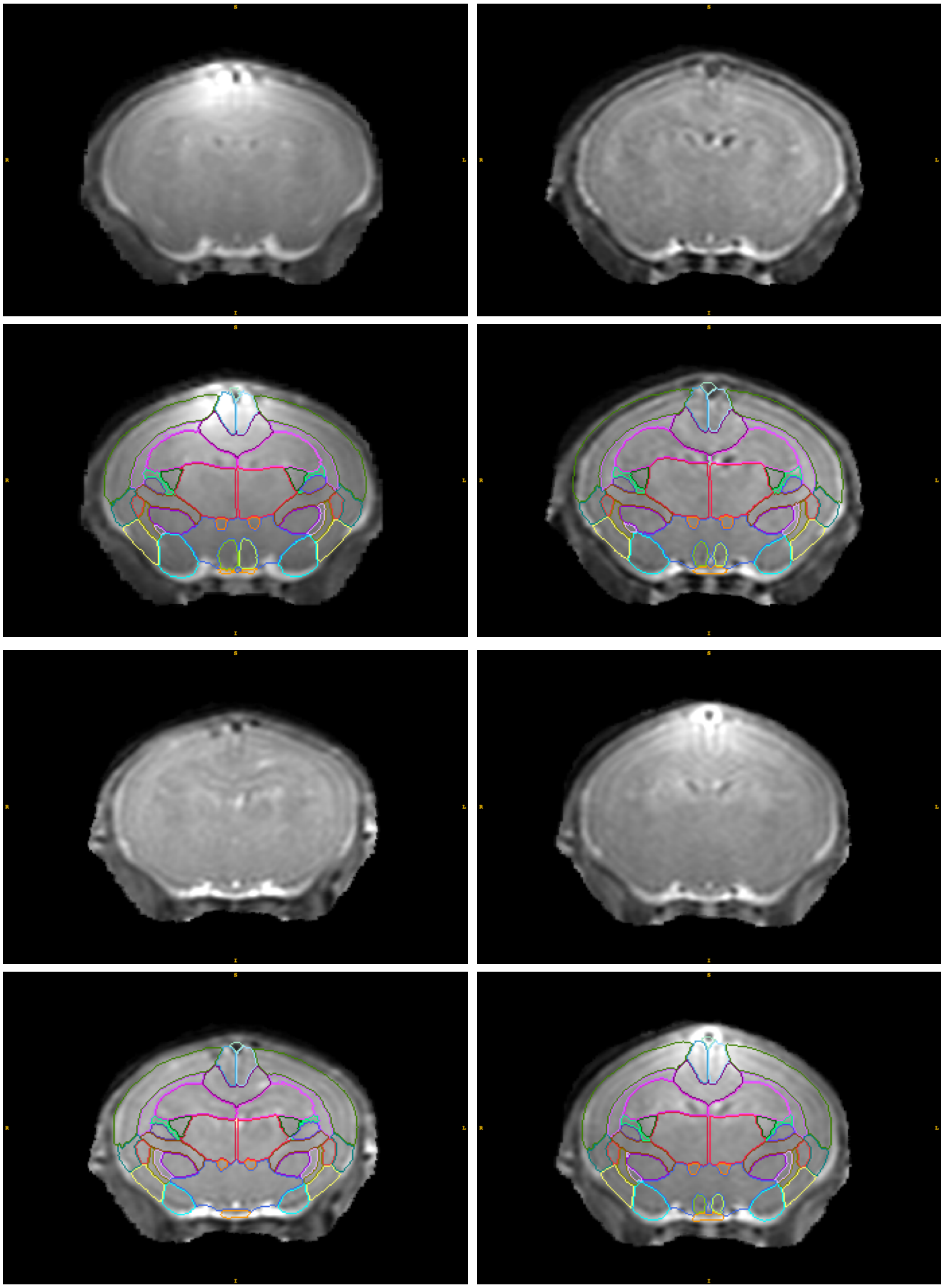

Figure 5: **Visual assessment for the in vivo automatic segmentation method.** The four subject with and without segmentation in coronal slice, after re-orientation, masking of the region of interest and resampling in the stereotaxic coordinate (top left first, top right second, low left third, low right fourth).
