# Supplementary material for: A magnetic resonance multi-atlas for the neonatal rabbit brain
Source: Neuroimage. 2018 Oct 1;179:187–98. doi: 10.1016/j.neuroimage.2018.06.029 (PMC6203700; doi:10.1016/j.neuroimage.2018.06.029)
Supplement: supplementary_material_E [file mmc5.pdf]

Appendix E: Additional validation scores

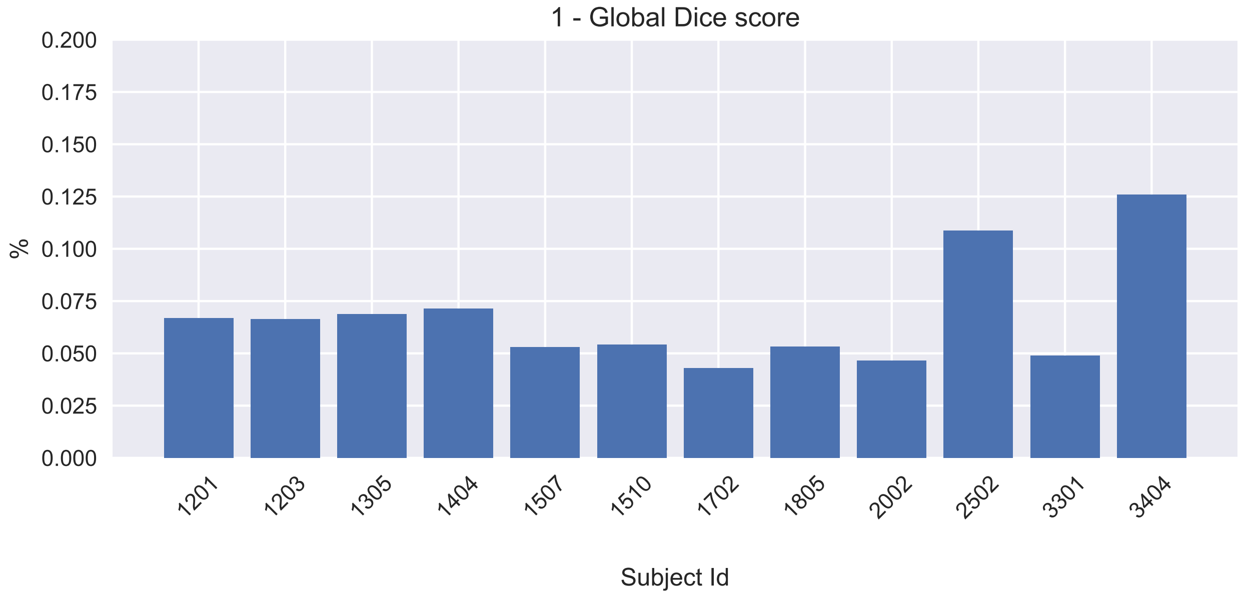

Figure 1: **One minus Global Dice Score between the manual and the automatic in the leave-one-out cross-validation experiment for each subject.** Global dice score between two segmentation is defined as the number of voxels where the segmentations are correctly aligned, divided by the average number of segmented voxels in the two segmentations. On the x-axis is reported the subject Id. The subjects 3403 and 2502 have visually more artefacts compared to the other subjects. This graph provides an indirect quantification of this visual assessment.

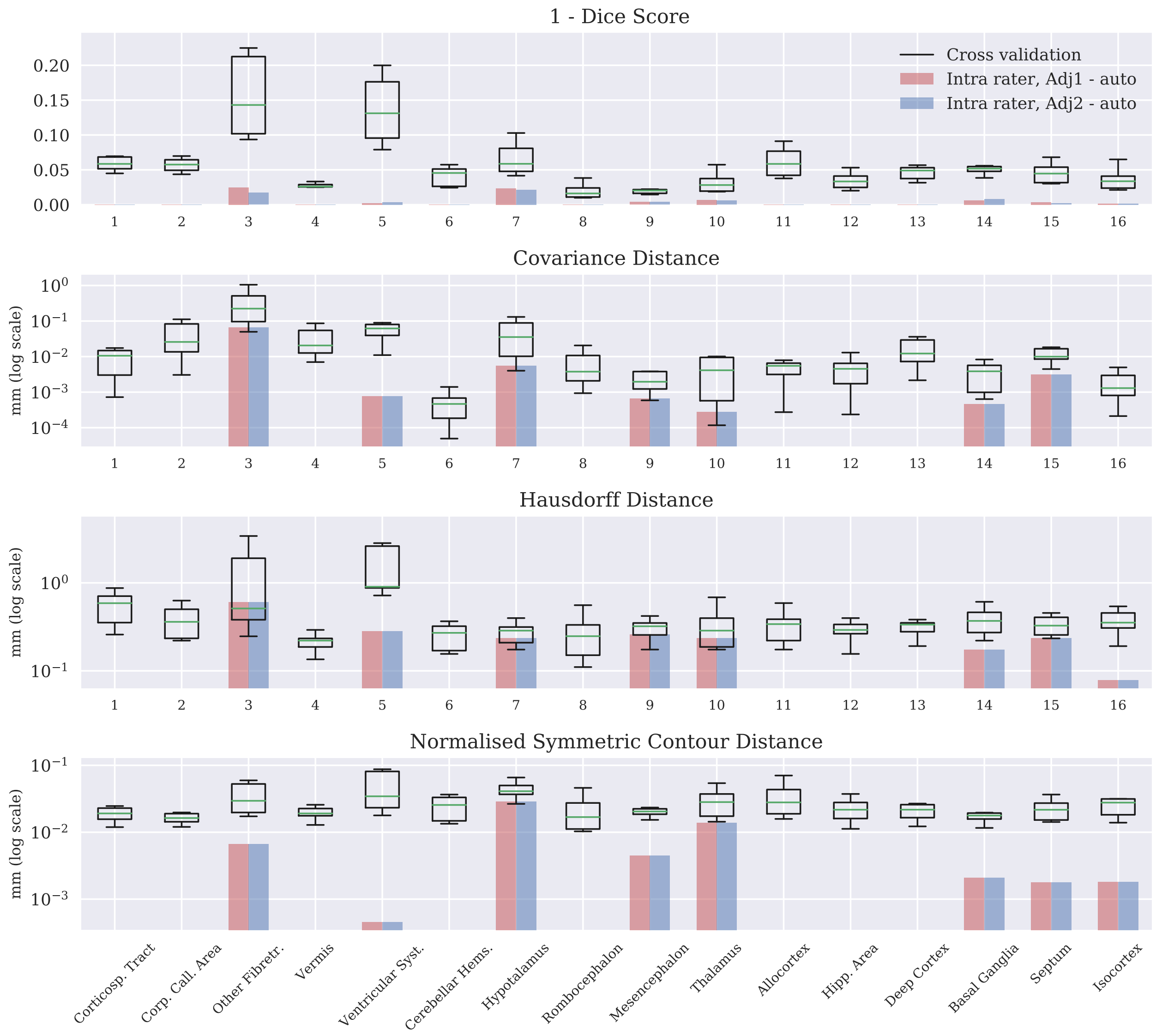

Figure 2: **Leave one out cross validation visualised above the intra-rater test-retest for the selected macro-regions.** This figure combines the results of the leave one out cross validation scheme and the intra-reater test-retest. Each point in the boxplot corresponds to the error at the given macro-region and for the given scoring system between the manual ground truth and the propagation of one of remaining 11 subjects. Differences between segmentations are scored with Dice score, covariance distance, Hausdorff distance and normalized symmetric contour distance. The correspondence between the x-axis and the macro-regions labels is reported in Table 2 in the paper. A randomly chosen segmentation underwent two times manual adjustment by rater$_1$ after the initial automatic segmentation. The intra-rater manual adjustment variability for each of the macro regions is shown in the bars. The left bars report the difference between the automatic and the first manual adjustment. The right bars report the difference between the automatic and the second manual adjustment. Only the regions belonging to the macro-regions 3, 5, 7, 9, 10, 13, 14, 15 and 16 required manual adjustment. The comparison between the two experiments reveals that the distances between the automatic segmentation and the manual segmentation is higher than the distance between the automatic method and a manual re-adjustment of the leave-one-out automatic segmentation.
